# Age‐dependent reduction in voltage‐gated inward sodium current and *Scn8a* gene expression in murine stellate ganglia

**DOI:** 10.1111/nyas.15298

**Published:** 2025-02-25

**Authors:** Bonn Lee, Shiraz Ahmad, Charlotte E. Edling, Christopher L.‐H. Huang, Fiona E. N. LeBeau, Kamalan Jeevaratnam

**Affiliations:** ^1^ School of Veterinary Medicine, Faculty of Health and Medical Sciences University of Surrey Guildford UK; ^2^ Physiological Laboratory University of Cambridge Cambridge UK; ^3^ Department of Biochemistry University of Cambridge Cambridge UK; ^4^ Biosciences Institute, Faculty of Medical Sciences University of Newcastle Newcastle upon Tyne UK

**Keywords:** aging, loose patch clamp, Nav 1.6 channel, Scn8a, stellate ganglia

## Abstract

Stellate ganglia (SG) provide sympathetic innervation to the heart and may predispose the myocardial conducting system to arrhythmias. However, little is known about age‐related changes in the electrophysiology of murine SG. We investigated alterations in the electrophysiological properties of SG with aging. The loose patch clamp technique was adapted to SG tissue to investigate the voltage‐gated ionic currents in its neuronal cells. We compared SG and ventricular cells from young (4 months) and aged (13 months) C57BL/6J mice to explore age‐related alterations in their voltage‐gated ionic currents (*n* > 30 patches, eight mice in each group). We observed that the voltage‐gated inward sodium current (peak *I*_Na(Max)_) was significantly decreased with aging in the SG, but not in the ventricle. Additionally, *Scn8a* gene expression, which encodes the Nav 1.6 channel, was decreased with aging in the SG. Application of loose patch clamp electrophysiology thus suggests that ionic current alterations with age in murine SG could contribute to cardiac autonomic dysregulation in geriatric conditions.

## Graphical abstract

An investigation into the electrophysiological properties of murine ventricular myocardium and stellate ganglia was conducted using the loose patch clamp technique at two distinct age points to assess aging‐mediated alterations in ionic currents. The voltage‐gated inward sodium current, quantified as maximum sodium current (*I*_Na(Max)_), was reduced with age in the stellate ganglia (SG) but not in the ventricular myocardium. RNA sequencing revealed differences in sodium channel gene expression between the heart and SG. *Scn8a* mRNA expression was decreased in SGs from 13‐month‐old mice compared to 4‐month‐old mice.

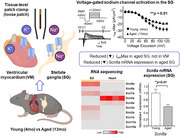

## INTRODUCTION

The stellate ganglia (SG) consist of interconnected clusters of sympathetic neuronal cell bodies which provide autonomic innervation to the heart.^1^ Previous research had focused on possible cardiac involvement of the autonomic nervous system (ANS) to establish empirical and mechanistic links with cardiac dysautonomia and disorders such as arrhythmias, chronic cardiac failure, and sudden cardiac death (SCD).^2^ Importantly, structural and functional changes in the SG are associated with cardiac dysautonomia, and dysregulation of the cardiac sympathetic input can predispose someone to SCD.^3^, ^4^, ^5^ Patients suffering from arrhythmic SCD demonstrated severe inflammation in their SG.^5^, ^6^, ^7^ The latter may result in potential dysregulation of cardiac sympathetic outflow.^6^, ^7^, ^8^ Additionally, animal studies have associated cardiac disease with structural and molecular remodeling of the SG. Thus, rats with induced chronic heart failure showed decreased mRNA expression of Cav2.2 channels, N‐type calcium current densities, and cell excitability in their cardiac postganglionic nerves.^9^ The porcine myocardial infarction model shows a decreased neuronal size and altered neurotransmitter reactivity of the cells in the SG.^10^ This evidence may associate molecular and cellular alterations in the cardiac sympathetic network with a range of cardiac pathologies.

Aging is considered the major risk factor for cardiovascular disease. In the aged individuals, 65 years or older, cardiovascular disease is the leading cause of death, resulting in 40% of all deaths.^11^ Aging affects neurocardiac regulation.^12^ In an age comparison study, older human subjects showed impaired noradrenaline release, higher resting plasma noradrenaline concentrations, and lower cardiac uptake of noradrenaline during transient exercise.^13^ Additionally, the aging in vivo murine model showed a decreased cardiac contractile response to beta‐receptor stimulation.^14^ Such age‐mediated autonomic dysregulation in the heart could predispose someone to arrhythmia or heart failure.^12^, ^15^, ^16^ In addition, the prevalences of cardiac dysautonomia disorders, such as orthostatic hypotension and SCD, increases with age.^11^, ^17^, ^18^ We hypothesized that aging might alter electrophysiological function in the cardiac ANS via the SG. It has been established that the SG undergoes postnatal morphological and functional reorganization after birth in murine animal models.^19^ However, few studies have assessed the long‐term changes in murine SG, and no study has focused on potential electrophysiological alterations with aging.

Cardiac autonomic regulation has attracted attention in relation to its possible relationships with cardiac electrophysiological abnormalities, such as atrial fibrillation, ventricular tachycardia, and SCD.^20^, ^21^, ^22^ Electrophysiological studies of the SG remain challenging as it is difficult to isolate cells from the SG. This makes electrophysiological interrogation using the conventional whole‐cell patch clamp technique difficult. We here used the loose patch clamp technique. Given the nature of how SG are excised, a loose patch clamp approach that enables exploration of tissue‐level electrophysiology becomes useful; its application permits minimally invasive techniques to preserve the tissue.^23^

The loose patch clamp uses a relatively large diameter (20–30 µm inner diameter) pipette, allowing the investigation of large areas of relatively intact tissue.^24^, ^25^ Earlier electrophysiological studies on the SG studied excitatory postsynaptic potentials by microelectrode recordings in the thoracic sympathetic chain,^26^ or applied whole‐cell patch clamp to isolated ganglionic cells.^27^ However, there have been no previous studies of voltage‐gated ionic currents in intact SG tissue. Voltage‐gated ionic currents in neuronal tissue reflect ion influx and efflux through voltage‐gated sodium and potassium channels, which are important to neuronal excitability.^28^, ^29^ Dysfunction of neuronal voltage‐gated channels (channelopathies) can lead to dysregulated neural action potentials (APs) and abnormal neuronal excitation.^30^

This study investigates age‐related electrophysiological changes in the SG and cardiac tissues by using the loose patch clamp technique. The gating properties of voltage‐gated inward sodium currents and transient outward potassium currents in the SG and ventricles were analyzed, and were compared between young and aged murine cohorts. RNA sequencing and quantitative polymerase chain reaction (qPCR) were performed to investigate their corresponding mRNA expression.

## METHODS

### Animal experiments and ethics

Male wildtype C57BL/6J 4‐ and 13‐month‐old mice (Janvier Labs) were maintained in the Biomed Research Facility at the University of Surrey under controlled conditions (ambient temperature 23±2°C, 12‐h light cycle with lights turned on at 07:00 a.m. and off at 7:00 p.m.) with food and water supplied *ad libitum*. Animals were given 1 week to acclimatize to housing conditions before experiments were conducted. All animal treatments and procedures were approved by the Animal Welfare Ethical Review Body of the University of Surrey (NASPA‐1819‐25 Amend 1). All procedures were performed according to the Animal Scientific Procedures Act 1986 (UK) and the National Institutes of Health (NIH) Guide for the Care and Use of Laboratory Animals.

### Ventricular tissue dissection and Langendorff perfusion

Mice were euthanized by Schedule I cervical dislocation (as per the Animal Scientific Procedures Act 1986, UK). The abdominal cavity was opened, and the thoracic cavity approached via the diaphragm. The heart was harvested with the pulmonary cassettes and vessels and immediately placed in ice‐cold Krebs−Henseleit solution (KH; 108 mM NaCl, 25 mM NaHCO_3_, 4 mM KCl, 1.2 mM KH_2_PO4, 1 mM MgCl_2_, 1.8 mM glucose, and 2 mM sodium pyruvate, bubbled with carbogen gas and pH adjusted to 7.4) and transferred to the patch clamp room. The aorta was cannulated with a 22‐guage stainless steel cannula and the tip was sutured with 5‐0 braided silk thread. The cannulated heart was placed in a Langendorff perfusion system and underwent retrograde perfusion under constant flow (2.1 mL/min) with 75 mL of a KH solution to which 10 mM 2,3‐butanedione monoxime (BDM) and 10 mM blebbistatin were added to give a KH‐BDM/blebbistatin solution to achieve electromechanical uncoupling.^31^ Then, the heart was placed into the KH solution for dissection of the right ventricle from the rest of the heart. The right ventricle was mounted on a Sylgard plate (SYLGARD 184 Curing agent, Cat# 761028, Sigma‐Aldrich) and placed in the patch clamp bath. The bath was filled with KH solution, and its temperature was maintained at 27–28°C by circulating heated water through a glass coil in the chamber.

### **Advantages of the loose patch clamp technique for investigation of the** SG

**FIGURE 1:**
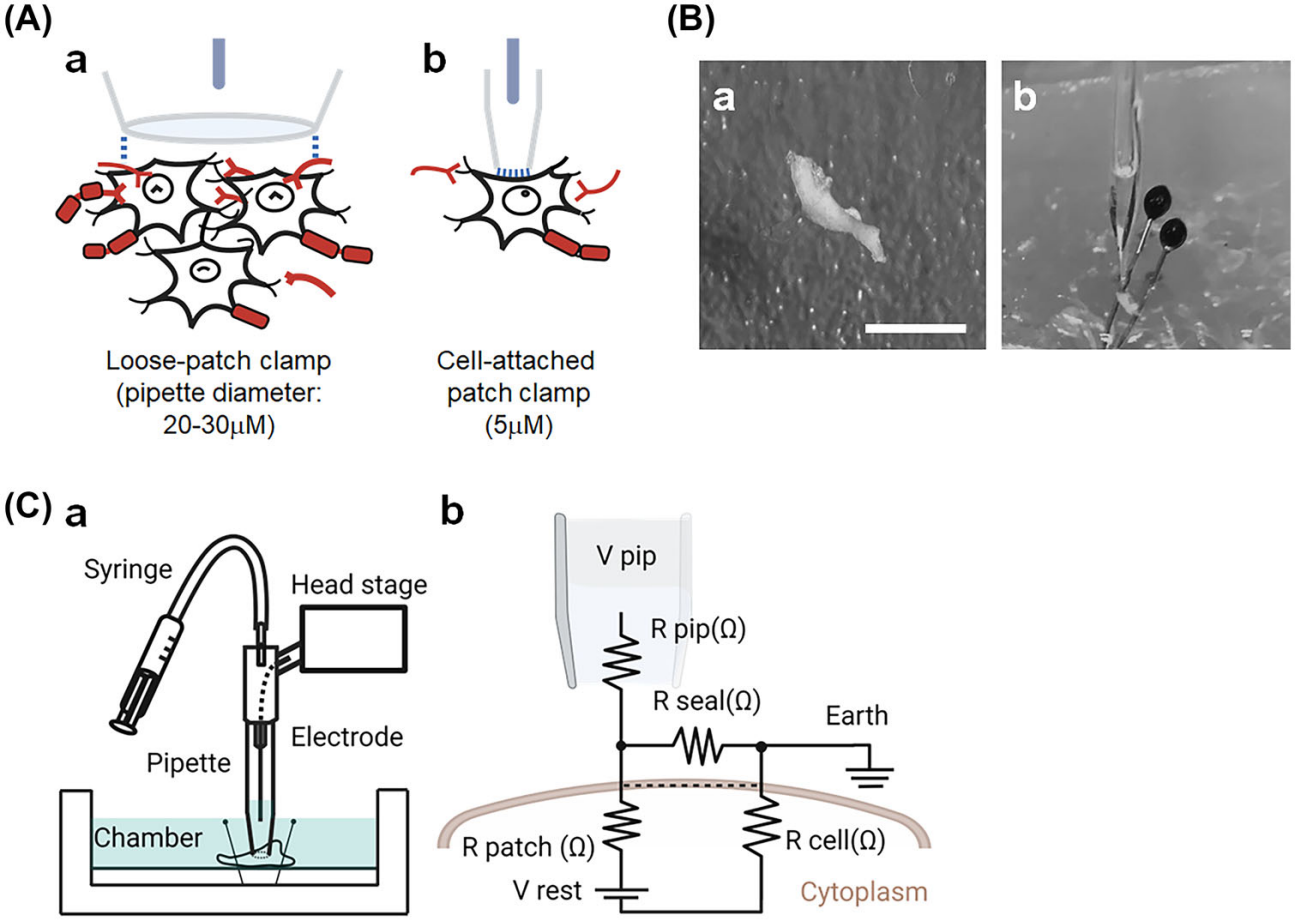
Loose patch clamping of murine stellate ganglia. (A) Schematics of patch clamp seal for the loose patch clamp and cell‐attached patch clamp; (a) patch clamp seal of the loose patch clamp; (b) cell‐attached patch clamp. (B) Patch clamp preparation for stellate ganglia; (a) microscope view of stellate ganglia; white scale bar, 2 mm; (b) stellate ganglia under the patch clamp pipette. (C) Schematic of the loose patch clamping stellate ganglia; (a) the configuration for the patch clamp bath and the pipette; the stellate ganglia were pinned on the bottom of the recording chamber, the headstage moved downward toward the tissue by maneuvering the manipulator, then the glass pipette attached perpendicularly onto the tissue surface; (b) equivalent circuit diagram for a loose patch clamp electrode on the tissue. Pipette clamped at voltage (*V*_pip_). Compensation for the voltage error arising from currents flowing through the series combination of the pipette resistance (*R*_pip_) and the seal resistance (*R*_seal_) was achieved using a bridge circuit in the custom‐designed loose patch clamp amplifier. The dashed line represents the tissue membrane. Resistance of the membrane patch (*R*_cell_). As the loose patch clamp technique alters the extracellular potential under the pipette‐patched seal area relative to the resting membrane potential (RMP), negative and positive voltage excursion in *V*_pip_ produce hyperpolarizing and depolarizing voltage steps relative to RMP, respectively. Cytoplasmic voltage relative to bath voltage (*V*_rest_). Illustrations created with Biorender.com.

To investigate the electrophysiology of the murine SG, a loose patch clamp system was used, an approach which was minimally invasive and preserved tissue integrity. The loose patch clamp does not require a conventional patch involving establishing a high‐resistance membrane seal. It thus allowed us to make membrane patches in cells within relatively intact tissue, while the whole‐cell patch clamp (conventional patch) requires a cell isolation procedure to form conventional seal patches (Figure 1A). The loose patch clamp recording technique has been previously employed in the rat pituitary,^24^ murine hippocampal slice,^32^ skeletal muscle,^33^ atrial,^34^ and ventricular^31^ tissue to investigate the characteristics of ion channels in intact tissue.

### **Dissection of** SG **and capsule removal**

After isolating the heart, the thoracic cavity was placed in an ice‐cold physiological solution (92 mM NaCl, 2.5 mM KCl, 30 mM NaHCO_3_, 1.25 mM NaH_2_PO_4_, 20 mM HEPES, 25 mM glucose, 10 mM MgCl_2_, 0.5 mM CaCl_2_; bubbled with carbogen gas; pH adjusted to 7.4) and moved to the loose patch clamp room. The SG were dissected using micro scissors and micro tweezers by a previously described technique.^35^ Right SG were used for the loose patch clamping. The SG tissue was then incubated at 36°C for 10 min in physiological solution to which 2 mg/mL collagenase P (Cat# COLLP‐RO, Roche Ltd.) was added in order to remove fat debris surrounding the SG (Figure S1A). We noted that this brief collagenase incubation did not alter the shape of the SG (Figure S1B). Then, the SG preparation was mounted on a Sylgard plate with standard insect pins (diameter 0.25 mm), and the plate was placed into the patch clamp chamber (Figure 1B). The recording chamber was filled with physiological solution and its temperature was maintained at 27–28°C.

### Pipette fabrication for the loose patch clamp

Pipettes for the loose patch were pulled from borosilicate glass capillary tubing (Cat# GC150‐10; Harvard Apparatus) by using a pipet puller (Cat# PC‐10 Narishige). The procedure for fabricating loose patch pipettes is similar to the two‐step process for making a conventional patch pipette; high heat to produce a narrowing of the pipette to a diameter of 300–400 µm, then a second pulling at a lower heat. The pipette tip was visualized under a microscope at 250× magnification, and the distal end of the pipette was scratched with a tungsten carbide knife (Cat# 71019‐10, Electron Microscopy Sciences) to form a small groove. Then, a force was applied to the distal end of the groove to break it off perpendicularly to the pipette axis, leaving an external end of the tip with a diameter of 20–30 µm. The pipette was then fire‐polished by using a micropipette fabricator (Cat# MF‐900, Narishige). Tips with an internal diameter of 18–26 µm after polishing were selected for experimental use. The pipette was placed on the microelectrode holder (Cat# Q45W‐B15P, Warner Instruments) incorporating an Ag/AgCl half‐cell connected to the headstage.

### Pipette positioning and forming a loose seal onto the tissue surface

By manipulating the syringe, the distal third of the pipette was filled with physiological solution. Then, the pipette was lowered perpendicular to the membrane of the SG using a fine vertical manipulator (Prior Scientific Instruments). Under a loupe, the pipette was positioned toward the cardiovascular pole, where ganglionic ion channels are abundantly expressed.^36^ The pipette tip was advanced until it made contact with the tissue surface. The contact with the tissue surface was recognized when it elicited a rise in the resistance at the pipette tip, as observed by a deflection in the oscilloscope trace, then the *R*_seal_ was compensated by adjusting the corresponding resistance in the compensating bridge circuit. To stabilize the seal, negative pressure was applied by gently withdrawing the syringe. The chamber was actively grounded at the reference potential to complete the circuit. Ag/AgCl electrodes were used to provide a reversible electrical connection between the chamber and the electronic circuit. The configuration of the pipette, electrode, and recording chamber is depicted in Figure 1C‐a.

### The loose patch clamp set up

The loose patch clamp uses low‐resistance (<1 megohm, MΩ) pipettes to electrically isolate a patch of membrane within the pipette end from the external solution with a low‐resistance seal (Figure 1C‐b). Figure 1C‐b depicts the electrical components of the loose patch seal at the pipette/membrane interface. The low resistance of the loose patch seal does not require extremely close apposition of the pipette and the membrane, which leaves the membrane and its ion channels in a more physiological state. However, the low seal resistance of the loose patch allows a large leak current to flow across the seal, thus a greater proportion of the total voltage drop to ground occurs over the resistance of the pipette. To maintain the tip of the pipette at the desired potential, the voltage clamp must, therefore, supply greater amounts of compensatory current. The low resistance of the seal adds significant noise due to the random movements of ions through the seal and provides a significant shunt to ground for active patch currents.

### General principles of loose patch clamp recording

The current flowing across the patch of membrane drawn into the pipette could be measured by recording the electrode in the pipette relative to the actively grounded reference potential of the chamber. The potential across the membrane within the patch then corresponds to the cell resting membrane potential (RMP) prior to the application of the pulse protocols. Then, the pulse protocols clamped the voltage of the fluid within the pipette through their sequence of command potentials. This achieves the required changes in potentials across the membrane within the patch. As the voltages are applied from the extracellular rather than the intracellular space, a negative voltage step causes hyperpolarization and a positive voltage step causes depolarization of the membrane patch relative to RMP. Membrane potentials in this paper are thus described relative to the RMP, and imposed voltage changes are described as changes in intrapipette potential.

### Chemicals and applications of channel blockers

Chemical reagents used were purchased from Sigma‐Aldrich (Merck Life Science) unless otherwise stated. The sodium channel blocker lidocaine (500 µM; Cat#L7757‐25G, Sigma‐Aldrich) and potassium channel blocker 4‐aminopyridine (500 µM 4‐AP; Cat#0940, TOCRIS) were used to achieve blocking of selected channels within the patched membrane. The channel blockers were dissolved in a physiological solution to prepare a working solution. For baseline recording, the patch clamp chamber was filled with a fresh physiological solution; the chamber was then replaced with the test solution (containing lidocaine or 4‐AP). Due to the nature of the loose patched seal, the membrane area under the patch is exposed to the external biochemical environment within the pipette. Twenty‐five min of equilibration time was allowed after replacing the solution. The solution was used up to a maximum of 40 min to prevent potential metabolite accumulation from the tissue preparation.

### Loose patch clamp recording and pipette size correction

An IBM computer driven by custom‐written software was used to deliver voltage clamp steps relative to the RMP. To detect the presence of ion channels in the membrane patch being investigated, depolarizing pulses to (RMP + 80) mV were applied. The details of the pulse protocols are described in the Results section. Currents were sampled at a 50 kHz digital sampling rate and filtered at a DC−10 kHz bandwidth using a 10 kHz Bessel low pass filter. Patches containing sodium channels produced kinetically characteristic inward currents. We investigated their voltage‐dependent activation and inactivation properties, as well as the voltage‐dependent activation of transient outward potassium currents. The currents obtained were normalized to the area of pipette tip to achieve the current density, following the formula: current density (pA/µm^2^) = current measured (nA) × 1000/π × [pipette radius (mm)].^2^

### Determination of channel opening kinetics by curve fitting to the Boltzmann equation

Each current−voltage curve was fit to the Boltzmann equation to calculate the opening kinetics for voltage‐gated channels. The Na^+^ currents (*I*) were related to the activating voltage *V*  =  *V*_1_ by a Boltzmann function expressed with the following equation: *I*  =  *I_(_*_max)_{1 − 1/{1 + exp [(*V* − V*)/*k*]}}. Here, the maximum value of the current is denoted by *I*_(Max)_, the voltage at half‐maximal current, by V*, and a coefficient describing the slope factor related to the voltage sensitivity of the current, by *k*. The slope factor is expressed in units of potential (mV), with a larger value denoting a shallow curve.

### Pulse protocol for investigating voltage‐gated sodium and potassium channel properties for the ventricle

This study first investigated the SG using the loose patch clamp technique. The pulse protocol for the SG preparation is described with each data set (see Results). The ventricular loose patch clamp data are provided in the Supporting Figures. Sodium channel activation, sodium channel inactivation, sodium channel time‐recovery from inactivation, potassium channel activation, and potassium rectifying channel properties were investigated. The pulse protocols for examining ventricular tissue with the loose patch clamp technique have been described previously.^31^, ^34^

### RNA extraction and library preparation for RNA sequencing

The mice were sacrificed by cervical dislocation in accordance with the Animal (Scientific Procedures) Act 1986 (UK) and decapitated. Then, the heart and SG tissues were harvested immediately after death and the tissues were stored in RNAlater solution (Cat#: AM7020, Thermo‐Fisher Scientific). The whole heart was isolated, and the SG tissues were trimmed to remove fat debris. The total RNA was extracted by using the RNAqueous Micro Total RNA Isolation Kit (Cat#: AM1931, Thermo‐Fisher Scientific). RNA purity and concentration were measured by 18S/28S ratio and RNA integrity number (RIN) on 2100 Bioanalyzer with RNA 600 Nano Chips (Cat#: G2939BA, Agilent). Samples that met the quality parameter of RIN above 7.0 were selected for library preparation. Library preparation and sequencing were carried out at Novogene (UK) Company Limited using the Illumina platforms.

### Relative mRNA expression analysis by the R package DEseq2

Data analysis for RNA sequencing was conducted using R version 4.3.1 Bioconductor DEseq2 version 1.26.0 package.^37^ The raw mapped read count provided by Novogene was normalized by the linear regression model of DEseq2 to compare the mRNA expression level between the SG and the heart. The mRNA expression levels of all the voltage‐gated sodium channels were compared.

### Quantitative polymerase chain reaction

Reverse transcription of RNA from each sample was performed using a High Capacity cDNA Reverse Transcription Kit (Cat#:4638814, Applied Biosystems; Thermo‐Fisher Scientific) with a thermocycler. The primers used are listed in Table S1. Relative mRNA expression was calculated with the ΔΔCt method.^38^ *ActB* and *Hprt1* were used as the reference housekeeping genes.

### Data handling and statistical analysis

We investigated multiple sites on a tissue surface with the loose patch clamp. Each patch‐clamped site counted as a separate data point (*n* of 1). When we applied the patch clamp electrode to SG tissue from a different subject, it was counted as an independent experiment. We examined four sites in each tissue. In cases where fewer than four sites were examined due to the size variance of the tissue, the number of sites investigated is denoted in the figure legends. The number of replicates is also listed in the figure legends. The statistical significance analyses were performed using R 4.4.0. All data were analyzed by first applying the Kolmogorov−Smirnov normality test, and Student's *t*‐test for comparing the means. One‐way ANOVA, and *post hoc* Tukey honestly significant difference (HSD) test were used to determine the significance level among multiple unrelated groups.

## RESULTS

### **Pharmacological validation establishing transient inward sodium and outward potassium currents in** SG **under loose patch clamp**

**FIGURE 2:**
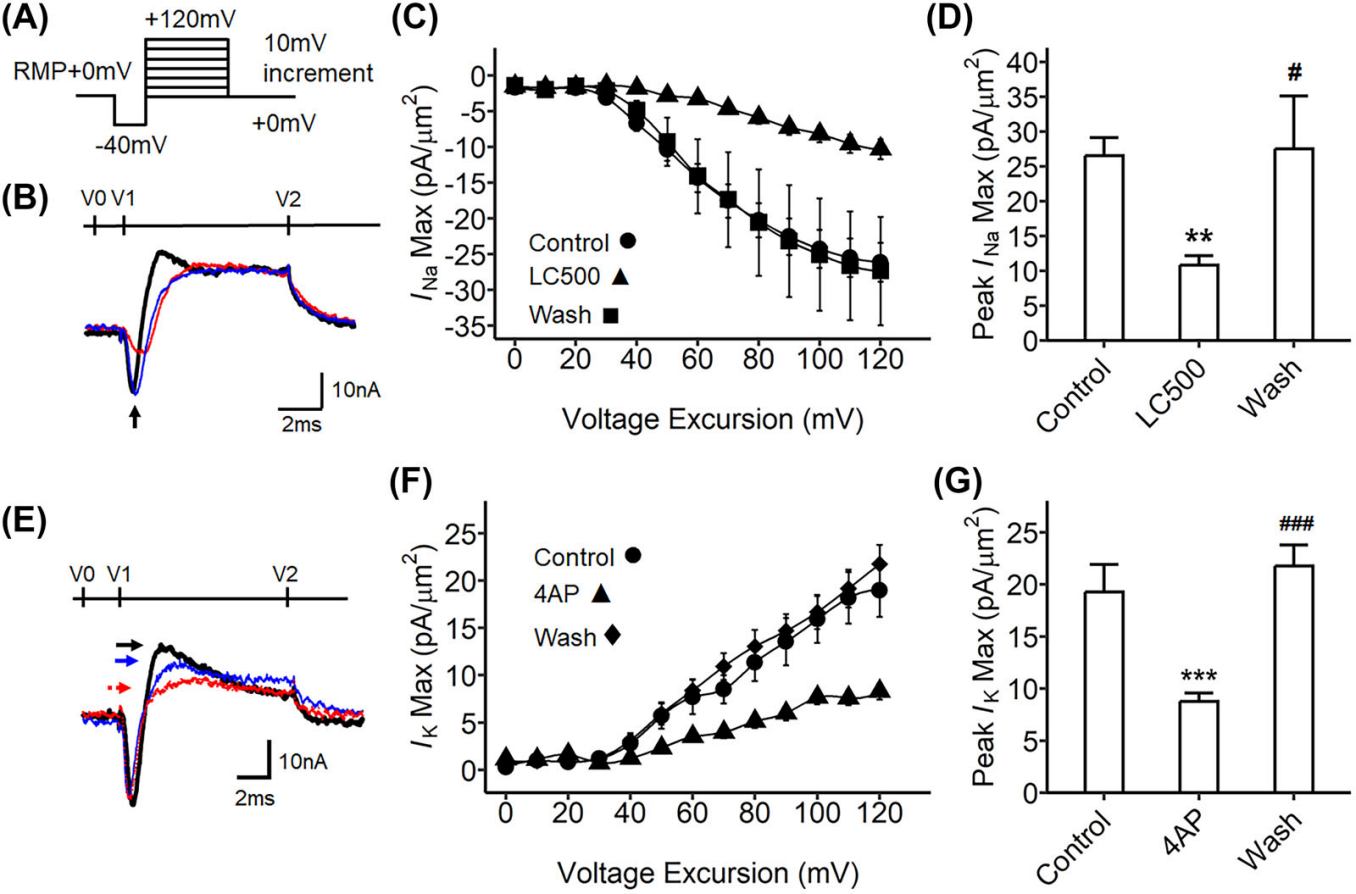
Pharmacological verification of inward sodium and transient outward potassium currents in stellate ganglia under the loose patch clamp. (A) Step pulse protocol to activate sodium currents for the stellate ganglia preparations. The pulse protocol began from the resting membrane potential (RMP). A 4‐ms duration prepulse was applied to remove residual current over the seal, then 10‐ms step‐increment pulses were applied to elicit the voltage‐gated currents from RMP−40 mV to RMP+120 mV; the end pulse was imposed at 10 ms. (B) Example current traces: black trace from recording with physiological solution control (control); red trace for lidocaine 500 µM in the physiological solution (LC500); blue trace for physiological solution washout of the lidocaine (Wash). The arrow points to the inward sodium on the trace (*I*_Na(Max)_). Tissues were collected from the 4‐month‐old mice. (C) The current–voltage curve for inward sodium currents; circle (physiological solution alone [Control], *n* = 12 patches, curated from three independent experiments, four different sites were examined per tissue), triangle (LC500, *n* = 12 patches), square (Wash, *n* = 8 patches). (D) The maximum *I*_Na(Max)_ in panel C: ANOVA, *F*_(2, 29)_ = 6.82, *p* = 0.003; Tukey HSD, ^**^ *p* = 0.009 physiological solution alone versus LC500, ^#^ *p* = 0.012 LC500 versus Wash. (E) Example current traces: black trace for physiological solution control; red trace for 4‐aminopyridine (4AP) 500 µM in physiological solution containing 4AP; blue trace for the preparation washout with physiological solution after applying 4AP (Wash); each arrow points to the transient outward current on the trace by the color. (F) The current–voltage curves for transient outward potassium currents; circle (physiological solution alone [Control], *n* = 7 patches, curated from three independent experiments, 2–3 sites were examined per tissue), triangle (4AP, *n* = 16 patches, curated from independent experiments, 4–5 sites were examined per tissue), diamond (Wash, *n* = 12 patches, curated from three independent experiments, four sites were examined per tissue). (G) The maximum *I*_K(Max)_ in panel F: ANOVA, *F* (2, 32) = 21.15, *p* = 0.0014, Tukey HSD, ^***^ *p<*0.001 physiological solution alone (control) versus 4AP, ^###^ *p* <0.001 4AP versus Wash. The error bars show the standard error of the mean.

Verification of the applicability of the loose patch clamp system to the SG preparation explored the actions of established sodium and potassium blockers (Figure 2A−D). The agents and concentrations used followed those of previous loose patch studies on brain slices.^24^, ^32^ First, the pan‐sodium channel blocker lidocaine was applied to the SG preparation studied under the loose patch clamp. A voltage step protocol was applied to elicit membrane depolarization (Figure 2A). Figure 2B illustrates examples of the current traces obtained. The inward current was reduced in the presence of lidocaine, and this effect was reversed following washout. This confirmed that the inward current was a lidocaine‐sensitive inward sodium current. Additionally, the maximum magnitude of the inward sodium current (*I*_Na(Max)_) was plotted against the applied voltage excursion (Figure 2C). The *I*_Na(Max)_ increased with increasing voltage excursion; its slope decreased as it approached its greatest investigated value at voltage RMP+110 mV. Figure 2D displays the peak *I*_Na(Max)_. Lidocaine (line with triangles) decreased the *I*_Na(Max)_ compared to its value in the physiological solution prior to the addition of lidocaine (*p* = 0.013). *I*_Na(Max)_ recovered upon washout with pure physiological solution (*p* = 0.04). Our loose patch system was thus applicable to measurements of the inward sodium current and its response to channel‐blocking agents in the SG.

**FIGURE 3:**
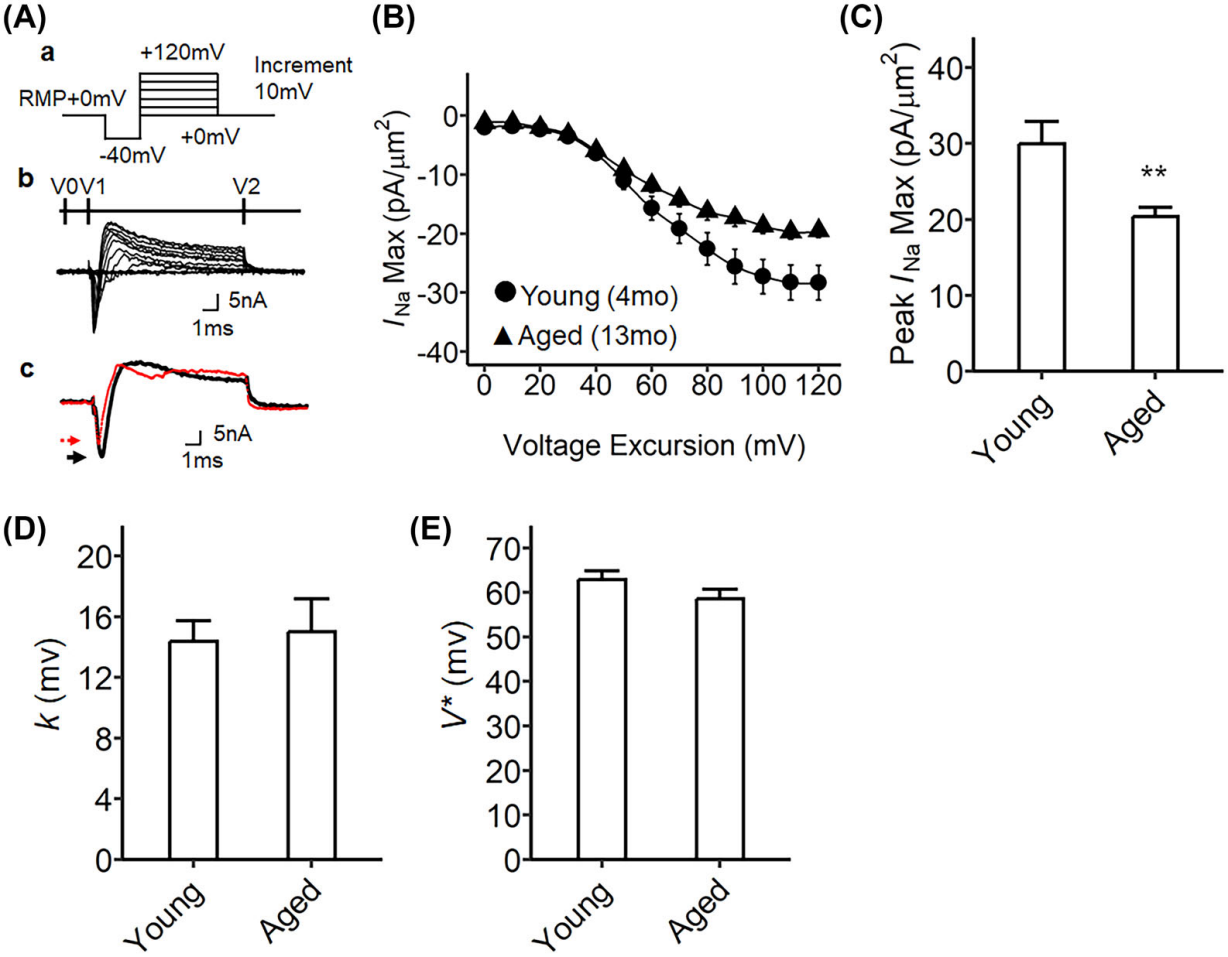
Activation properties of voltage‐gated inward sodium currents of murine stellate ganglia preparations from two different aging time points. (A) Step pulse protocol to activate sodium currents for the stellate ganglia preparations; (a) activation pulse protocol began from the resting membrane potential (RMP), a 4‐ms duration prepulse was applied to the patched area to remove any residual current over the seal area (V0), then 10‐ms step‐increment pulses were applied to elicit the voltage‐gated currents from RMP−40 mV to RMP+120 mV (V1). Then, the end pulses were applied at 10 ms from depolarization (V2). Shown are the family of current traces recorded from the step pulse protocol a: (b) the comparison of current traces for the young and aged preparations; (c) black, young (4‐month‐old) heart; red, aged (13‐month‐old) heart; the arrows mark the point of the greatest value of sodium inward current on the current trace (*I*_Na(Max)_), young (black) and aged (red). (B) The current–voltage curves for sodium channel activation, *I*_Na(Max)_, are plotted against the voltage excursion; young, stellate ganglia from 4‐month‐old time point (circle, *n* = 28, curated from nine independent experiments, 3–4 sites were examined per tissue); aged, stellate ganglia from 13‐month‐old time point (triangle, *n* = 26, curated from eight independent experiments, 3–4 sites were investigated per tissue). The peak *I*_Na(Max)_ increased as the voltage excursion up to the maximum peak *I*_Na(Max)_ at 100–110 mV, and the activation decayed at 120 mV. (C) A comparison of the peak *I*_Na(Max)_ in young and aged preparations, *t*_43.35_ = 0.162, **p‐value = 0.005. (D) The Boltzmann slope factor (*k*). The sodium activation current was fitted with the Boltzmann function, *k* of 13.5 mV for *I*_Na_ in young, 15.8 mV for *I*_Na_ in aged preparation, no significance. (E) The half‐maximal voltage (V*) from the Boltzmann equation derived from the plot in panel B, 60.99 mV for *I*_Na_ in young, 60.49 mV for *I*_Na_ in aged preparation: no significant difference. The error bars show the standard error of the mean.

The pan‐potassium channel blocker 4‐AP was next applied to the loose‐patched SG to test its effect on outward current responses (Figure 2A,E–G). These were also studied by step voltage protocols (as in Figure 2A). The transient outward current following the inward sodium current was measured (Figure 3E). 4‐AP eliminated the transient outward current (red trace in Figure 2E), but it did not affect the inward current. We thus identified the affected component as a transient outward potassium current. The maximum amplitude of the transient outward current (*I*_K(Max)_) from each voltage sweeps was plotted against voltage excursion (Figure 2F). The peak *I*_K(Max)_ value at RMP+120 mV was compared between physiological solutions before and following the addition of 4‐AP, and after 4‐AP washout (Figure 2G). The peak *I*_K(Max)_ with 4‐AP treatment was significantly reduced (*p*<0.001), and the transient outward current recovered on washout (*p*<0.001).

### **Aging affects sodium channel activation in the** SG **but not in the ventricles**

We investigated sodium channel activation currents in the SG studied under the loose patch clamp and compared two different cohorts: young and aged tissue from mice aged 4 and 13 months, respectively. First, sodium channel activation properties in the SG tissues were examined. A step voltage protocol was used to stimulate fast inward current (Figure 3A), and the amplitude of the inward sodium current was plotted against the voltage excursion (Figure 3B). In both young and aged SG, the *I*_Na(Max)_ reached its greatest value at the voltage RMP+110 mV. We observed that this greatest *I*_Na(Max)_ was significantly lower in the aged SG preparation compared to that in young mice (*p* = 0.005, Figure 3C). A current–voltage curve was fitted using the Boltzmann equation to compare the kinetics of sodium channel activation for young and aged SG. The slope factor (*k*) and the half‐maximal voltage (V*) were not significantly different between SG from young and aged mice (Figure 3D,E). We also investigated the ventricles from the same mice with the loose patch clamp, but there were no significant differences between ventricles from young and aged mice (Figure S2). In conclusion, the greatest value of *I*_Na(Max)_ at the steady state differed between young and aged SG, while the detailed characteristics of gating for sodium channel activation did not differ with aging.

### **Comparison of sodium inactivation properties in** SG **with age**

**FIGURE 4:**
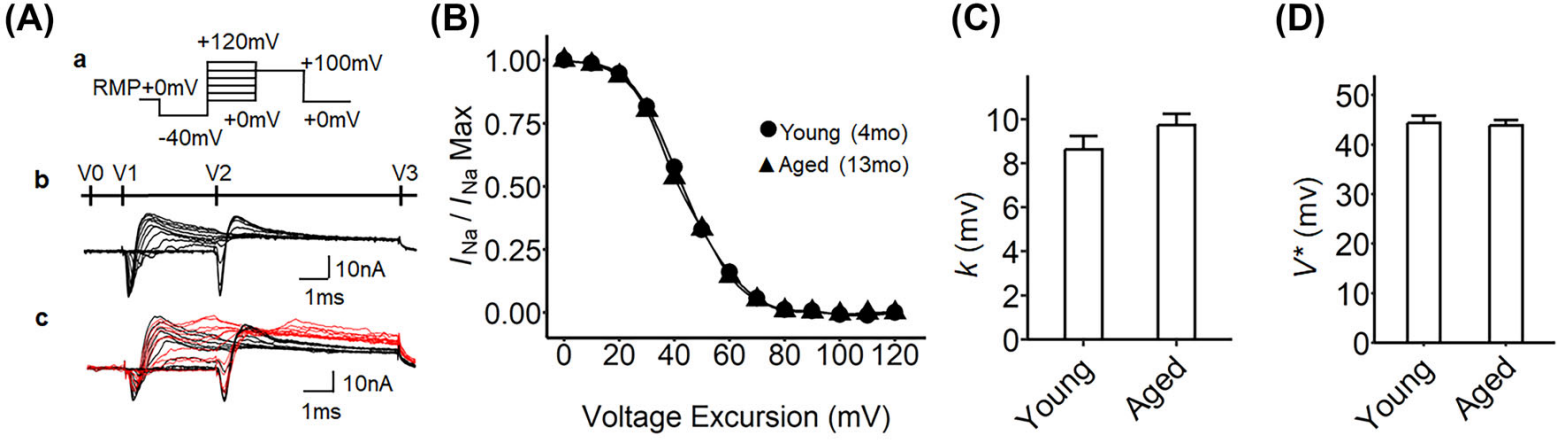
Inactivation properties of voltage‐gated inward sodium currents of murine stellate ganglia preparations from two different aging time points. (A) Current protocol and traces for activating sodium currents in stellate ganglia; (a) step pulse protocol activation began from the resting membrane potential (RMP), a 4‐ms duration prepulse was applied to the patched area to remove any residual current within the seal (V0), then 10‐ms step‐increment pulses were applied to elicit the voltage‐gated currents −40 mV relative to the RMP (RMP−40 mV) and to +120 mV relative to the RMP (RMP+120 mV; V1), then the voltage sweeps were stepped to a RMP+100 mV (V2); finally, the end pulses were applied at RMP+0 mV (V3); (b) a family of current traces from the sodium channel inactivation protocol in (a). V0, −40 mV prepulse at a 1‐ms duration; V1, 10 mV step‐increment pulses at a 5‐ms duration; V2, 100 mV depolarizing pulse at a 10‐ms duration; V3, end pulse at a 15‐ms duration; (c) representative current traces: young (4‐month‐old mice, black trace) and aged (13‐month‐old mice, red trace). (B) The current–voltage curves of sodium channel inactivation under loose patch. Patch clamp recording was conducted in physiological solution. Each sodium current (*I*_Na_) trace from voltage excursions was normalized to its maximum value (*I*_Na(Max)_) at RMP+0 mV; young (circle, *n* = 33, curated from nine independent experiments, 4–5 sites were examined per tissue); aged (triangle, *n* = 32, curated from eight independent experiments, four sites were examined per tissue). (C) The Boltzmann slope factor (*k*) for stellate ganglion in young and aged preparation. The sodium activation current was fitted with the Boltzmann function, giving *k* of 9.73 mV for *I*_Na_ in young preparation and 8.62 mV for *I*_Na_ in aged preparation. No significant difference. (D) The half‐maximal voltage (V*) from the Boltzmann equation derived from the plot in panel B, −43.86 mV for *I*_Na_ in young preparation, −44.40 mV for *I*_Na_ in aged preparation. No significant difference. The error bars show the standard error of the mean.

We next explored sodium current inactivation properties in the SG preparations from young and aged mice using the loose patch clamp method (Figure 4). The pulse protocol is illustrated in Figure 4A‐a. The prepulse voltage at the RMP was first stepped from the RMP to RMP−40 mV (V0 in Figure 4A‐b). Depolarizing steps were then applied to test voltages between RMP+0 mV and RMP+120 mV through 13 successive sweeps (V1). These depolarizing steps caused activation and then inactivation of sodium channels, with the degree of the inactivation determined by the voltage of the depolarizing step (V1). The degree of inactivation was then evaluated by subsequently applying a step to RMP+100 mV that caused sodium channel reactivation (V2), and the voltage sweep completed with a voltage step returning the membrane potential to RMP+0 mV (V3). The amplitude of the sodium current elicited by the second step to RMP+100 mV was considered to reflect the extent of sodium channel inactivation. Figure 4B plots the sodium channel inactivation against voltage excursion. The inactivation was expressed as the value of sodium current normalized to that at the second pulse from voltage RMP+0 mV to RMP+100 mV (Figure 4B). In the beginning (at RMP+0 mV), the sodium channel was 100% activated and then inactivated. The inactivation results were fitted to a Boltzmann curve to compare the kinetics of sodium channel inactivation between young and aged SG (Figure 4C,D). There were no significant differences in the extent of recovery, the slope factor (*k*), and V* with age. The sodium channel inactivation properties were also investigated in ventricular preparations in young and aged mice. Here, no significant age‐dependent changes were observed (Figure S3). Our data thus suggest that aging, therefore, did not alter sodium channel inactivation properties in both the SG and ventricle.

### **Decreased rates of time‐dependent sodium channel recovery in aged** SG

**FIGURE 5:**
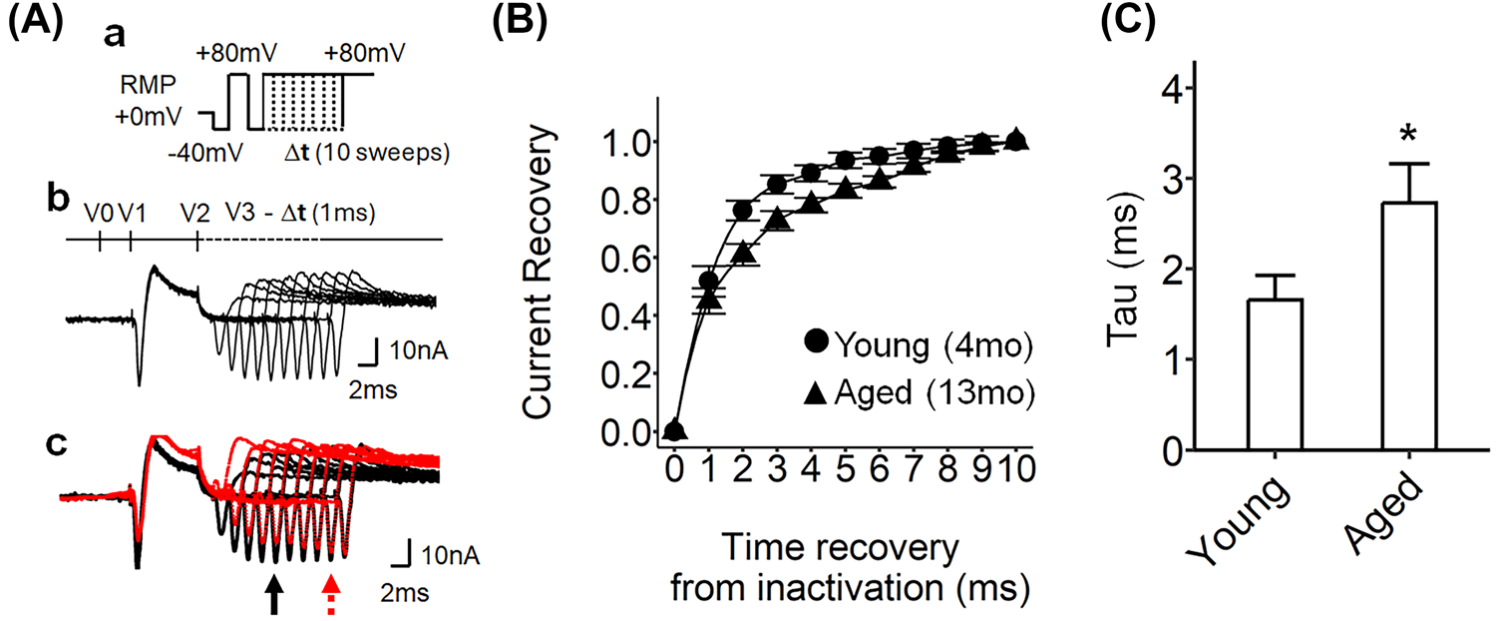
Time‐dependent recovery from inactivation of sodium currents following restoration of the membrane potential in murine stellate ganglia preparations from two different aging time points. (A) Pulse protocol and traces of currents; (a) the pulse protocol for time‐increment sodium channel recovery from inactivation: RMP+0 mV, 0 mV from the resting membrane potential (RMP), activation pulse protocol began from the RMP, a 4‐ms duration prepulse was applied to the patched area to remove any residual current over the area of the pipette (V0), then 5‐ms duration pulses were applied (RMP+80 mV) to activate ion channels and elicit the voltage‐gated currents (V1), then RMP−40 mV was applied to remove the residual voltage, the channels were predicted to inactivate during this phase. Then, an RMP+80 mV pulse was applied to reactivate the current (V3), the voltage sweeps were imposed with a 1‐ms time‐increment; (b) a family of current traces from the sodium channel inactivation protocol in (a). V0, RMP−40 mV prepulse at a 1‐ms duration; V1, RMP+80 mV pulse at a 5‐ms duration; V2, RMP−40 mV pulse at a 10‐ms duration; V3, RMP+80 mV pulse at different time intervals; Δ*t*, 1‐ms increment via the 10 successive sweeps making up the protocol; (c) the example traces for the young (4‐month‐old) and aged (13‐month‐old) stellate ganglia preparations, the arrows mark the point of the inward sodium current after the channel inactivation in young (black) and aged (red) preparations. (B) The time‐voltage curves of sodium channel recovery from inactivation, the peak *I*_Na_ was plotted against time between the termination of the conditioning and imposition of the test pulse; young preparation (circle, *n* = 22, curated from nine independent experiments, 2–3 sites were examined per tissue); aged preparation (triangle, *n* = 22, curated from eight independent experiments, 2–3 sites were examined per tissue); each peak *I*_Na_ was normalized by the *I*_Na_ peak at the termination time point (10 ms). (C) The time constant, 1.65 ms for *I*_Na_ in young preparation, 2.71 ms for *I*_Na_ in aged preparation; significance level for the comparison between young and aged preparation (*t*_40.70_ = 2.10, *p‐value = 0.04). The error bars show the standard error of the mean.

The time dependence of sodium channel recovery from voltage inactivation was interrogated in SG preparations (Figure 5). This was done by applying a pulse protocol examining the time course of recovery from inactivation. The pulse protocol began at the RMP and was followed by a prepulse step to RMP−40 mV. A voltage step of duration 5 ms to RMP+80 mV was then imposed to achieve sodium channel inactivation. The voltage was then stepped back to RMP−40 mV for 5 ms to terminate the inactivation. Finally, a depolarization step to RMP+80 mV was imposed following different time intervals (Δ*t*) to examine the refractoriness of the sodium channels after the prior channel activation (V3). In the representative traces in Figure 5A‐c, currents from aged SG (red traces) exhibited slower restoration in sodium channel activation compared to that in SG from young mice (black traces). The value of inward sodium current, normalized to the maximum value at 100% recovery, was plotted against the recovery time (Figure 5B). The time constant (τ) was calculated from Figure 5B to estimate the rate of change for the recovery (Figure 5C). The aged SG exhibited a significantly higher τ value than that of the young SG, indicating that the extent of sodium channel recovery from inactivation was slower (*p* = 0.04). Additionally, the age‐dependent changes in the sodium channel recovery time from inactivation were observed only in the SG and not in the ventricular preparation (Figure S4).

### Comparison of transient outward potassium currents in the SG from young and aged mice

**FIGURE 6:**
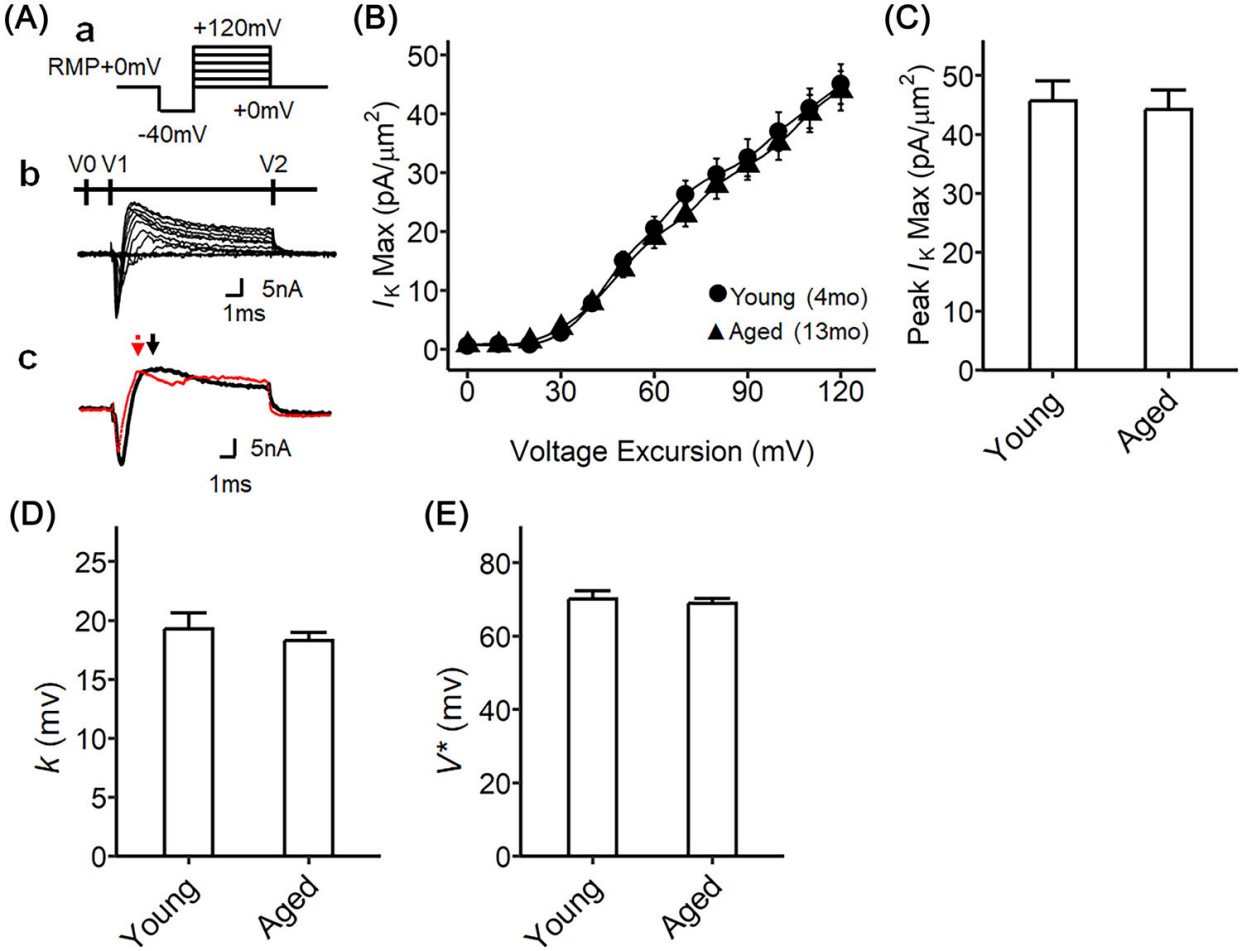
Activation properties of voltage‐gated transient outward potassium currents of murine stellate ganglia preparations from two different aging time points. (A) Step pulse protocol to activate sodium currents for the stellate ganglia; (a) activation pulse protocol began from the resting membrane potential (RMP), a 4‐ms duration prepulse was applied to the patched area to remove any residual current over the area of the pipette, then 10‐ms step‐increment pulses were applied to elicit the voltage‐gated currents from RMP−40 mV to RMP+120 mV; family of current traces recorded from the pulse protocol of (a); (b) an example of current traces from voltage excursions. (c) Black trace, young tissue preparation (4‐month‐old); red trace, aged tissue preparation (13‐month‐old); the protocol and data set are identical to that of investigating sodium channel activation (Figure 4); however, here, the transient outward current was analyzed; the arrows mark the points of transient outward potassium current for the young (black) and aged (red) preparations. (B) The current−voltage curves, *I*_K_ are plotted against the voltage excursion, young stellate ganglia of 4‐month‐old time point (circle, *n* = 28, curated from nine independent experiments, 3–4 sites were examined per tissue) and aged stellate ganglia of 13‐month‐old time point (triangle, *n* = 26, curated from eight independent experiments, 3–4 sites were examined per tissue). (C) The peak *I*_K(Max)_ at RMP+120 mV, 45.71 mV for *I*_K_ in young, 44.20 mV for *I*_K_ in aged preparation. No statistical significance. (D) The Boltzmann slope factor (*k*). The sodium activation current was fitted with the Boltzmann function, giving *k* of 19.28 mV for *I*_K_ in young, 18.29 mV for *I*_K_ in aged preparations. No significant difference. (E) The half‐maximal voltage (V*) from the Boltzmann equation derived from the plot in panel B, giving 70.14 mV for *I*_K_ in young, 68.99 mV for *I*_K_ in aged preparation. No significant difference. The error bars show the standard error of the mean.

We also analyzed the transient outward potassium current of SG from young and aged mice (Figure 6). We analyzed the maximum value of the fast outward current (*I*_K(Max)_) instead of the inward sodium current (black and red arrow in Figure 6A‐c). This value was plotted against the voltage excursion (Figure 6B). The peak *I*_K(Max)_ did not show significant changes with aging (Figure 6C). The current−voltage data was fitted using the Boltzmann equation (Figure 6D,E). The potassium currents did not differ between SG from young and aged mice, in contrast to corresponding findings bearing on the inward sodium current. Thus, only the sodium inward currents were affected by aging, while the rest of the trace was unchanged. This also confirmed the reliability of observations of altered sodium current, as the potassium current remained unchanged.

The potassium currents were investigated in loose patch‐clamped ventricular preparations from young and aged mice. The activation of potassium currents (Figure S5) and potassium rectifying currents (Figure S6) were investigated. No significant differences were detected in these ventricular potassium channel properties with aging. The voltage protocols for examining potassium currents in ventricular tissue with the loose patch have been previously described.^31^ The potassium currents similarly did not change with age in the ventricle.

### **Relative mRNA expression of major sodium channels in the heart and** SG

**FIGURE 7:**
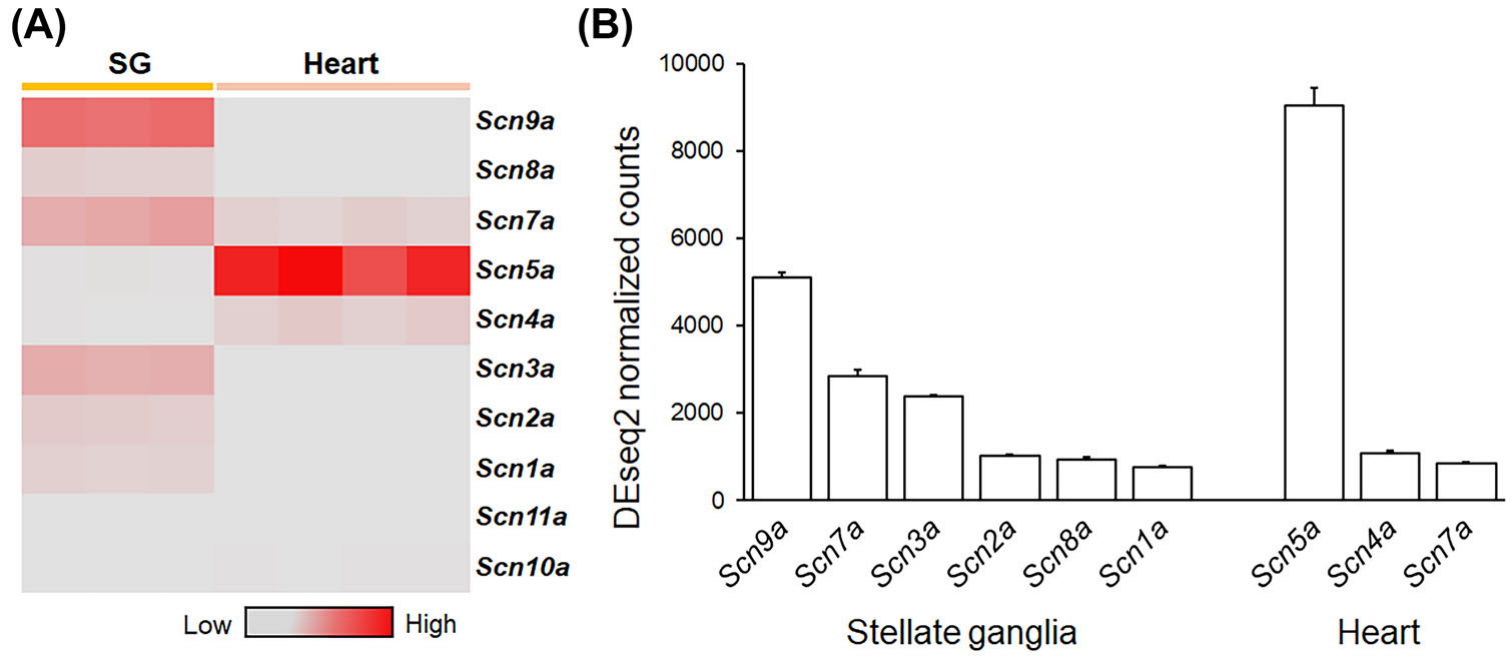
Relative mRNA expression levels of the major voltage‐gated sodium channels in the stellate ganglia and heart by RNA sequencing. The mRNA expression of the voltage‐gated sodium channels was investigated by RNA sequencing the stellate ganglia (*n* = 3) and heart (*n* = 4). (A) Heatmap illustrating the relative gene expression of voltage‐gated sodium channels in the stellate ganglia and heart; red represents higher gene expression, gray represents lower expression. (B) The DEseq2‐normalized counts of selected sodium channels from the heatmap. The DEseq2‐normalized counts were shown with a cut‐off level of 100 in DEseq2 unit count. The error bars show the standard error of mean. The stellate ganglia and heart were collected from 4‐month‐old male mice.

Our data thus showed age differentially affected the sodium currents in the SG but not the ventricle. This prompted a hypothesis attributing the different responses to aging in the SG and heart to differing voltage‐gated sodium channel expressions. Accordingly, RNA sequencing was conducted to profile sodium channel mRNA expression in the SG and heart (Figure 7). We compared mRNA expression levels of the voltage‐gated sodium channels by DEseq2 normalized counts. The heatmap provides an overview comparing different channel expressions between the SG and heart (Figure 7A). The major sodium channels in the SG (left) and the heart (right) are shown in the graph (Figure 7B), with a cutoff level of 100 (unit: DEseq2‐normalized counts). Differing sodium channel subtypes were expressed in SG and the heart. In the SG, *Scn9a* was the most abundantly expressed sodium channel, followed by *Scn7a, Scn3a, Scn2a, Scn8a*, and *Scn1a*. The heart expressed fewer sodium channel subtypes; *Scn5a* was the most highly expressed, followed by *Scn4a* and *Scn7a*. In contrast, the mRNA expression of *Scn5a* in SG was below the cutoff level of 100 (Figure 7B). This suggests that the SG and heart generate voltage‐gated sodium currents through different voltage‐gated sodium channel types. This may explain the changes of the voltage‐gated inward sodium current in the SG with aging but not in the heart.

### ***Scn8a* mRNA expression altered by aging in** SG

**FIGURE 8:**
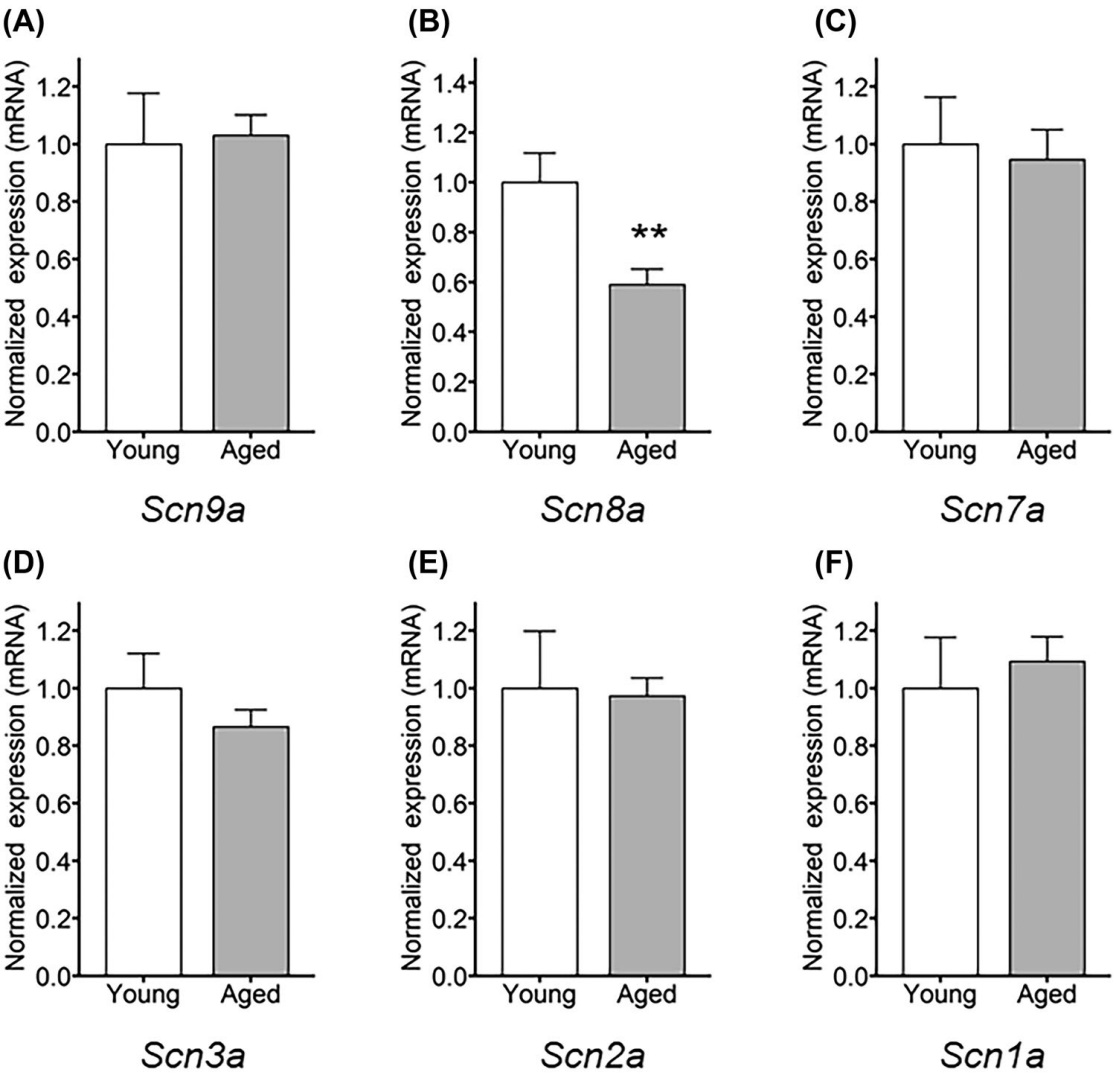
Real‐time qPCR confirmed the relative mRNA expression of the major voltage‐gated sodium channels in stellate ganglia from young versus aged mice. The mRNA expression levels of the sodium channels were investigated with real‐time qPCR. The relative gene expression levels were calculated by following ΔΔCq analysis method. Each Cq value was normalized by the mean Cq value of beta‐actin (*Actb*) and hypoxanthine‐guanine phosphoribosyl transferase (*Hprt1*) genes. The bar graph was normalized to express the geometric mean of the young group, represented as 1.0. *Scn8a*, *t*_12_ = 3.161, **p = 0.0082, young versus aged (*n* = 7 in each group). The error bars show the standard error of mean. Tissues were collected from 4‐month‐old mice for young, and from 13‐month‐old mice for aged.

Differential ion channel gene expression changes the current density in neuronal tissue.^39^ The relative level of mRNA expression of the major sodium channels was compared in SG from young (4‐month‐old) and aged (13‐month‐old) mice. (Figure 8; *n* = 7 in each group). The Cq values were normalized by those of beta‐actin (*Actb*) and hypoxanthine‐guanine phosphoribosyltransferase (*Hprt1*). We confirmed that these housekeeping genes were expressed at similar levels in the young and aged samples (Figure S7). The mRNA expression of *Scn8a* significantly differed between SG from young and aged mice (Figure 8B; *t*_12_ = 3.161, *p*‐value = 0.0082). Other sodium channels remained similar in SG from aged and young mice. The result showed changes in mRNA expression of *Scn8a* with aging in SG.

## DISCUSSION

This study explored electrophysiological characteristics of aging for the heart and the SG. Sodium and potassium currents from the SG and ventricular tissue preparations were investigated by using the loose patch clamp technique. Additionally, the major sodium channels expressed in the SG and heart, and their changes with age, were investigated at the mRNA level. This investigation has made three novel findings. First, we successfully established a method for examining the tissue‐level current in SG tissue by applying the loose patch technique. Second, we demonstrated that the channel opening properties of voltage‐gated sodium but not potassium channels show age‐dependent changes in SG but not in ventricles. Third, we report the quantitative mRNA expression of sodium channels in the murine SG and heart, and *Scn8a* was differentially expressed at the mRNA level between young and aged mice.

This study is the first to our knowledge to apply the loose patch clamp recording approach to murine SG tissue. We established the loose patch as an approach to examine altered ion channel properties in the SG in pathological conditions. Recent studies have suggested that alterations in SG function could reflect molecular or electrophysiological alterations in the heart.^6^, ^40^ The loose patch clamp technique enabled us to record the voltage‐gated sodium and potassium currents from the SG tissue, avoiding invasive cell isolations. Here, we conducted an electrophysiological investigation in SG by comparing SG and ventricles to examine the physiological changes associated with aging in young and aged wildtype C57BL/6J mice. Admittedly, we here used 13‐month‐old middle‐aged rather than geriatric mice, but could nevertheless focus on age‐dependent electrophysiological changes.^41^

Second, this study reports that the activation and time‐recovery properties of the inward sodium current in SG were different between young and aged mice but potassium currents were not changed. In contrast, in the ventricular preparations, aging did not affect either sodium or potassium currents. Consequently, aging seems to affect the voltage‐gated sodium channels in a tissue‐dependent manner. Nonetheless, it has not been confirmed that the reduction in inward sodium current density results in a functional or pathological change in the SG. Furthermore, the alteration in sodium channel properties was restricted to the peak *I*_Na(Max)_, and the kinetics and voltage dependence of the sodium channel gating did not differ between the age groups. The differences in the greatest values of *I*_Na(Max)_ could arise from different sodium channel densities in SG cells of young and aged SG mice. It could also reflect heterogeneities between SG neurons, including neuronal subtype variations within murine sympathetic cholinergic ganglia.^42^ Sharma et al. reported that the AP threshold can differ in SG cells depending on their neuropeptide expression.^36^

Third, this study revealed that *Scn8a* mRNA expression decreased with aging in SG. The *Scn8a* gene is known to be widely expressed in the nervous system and encodes Nav1.6 voltage‐gated sodium channels. *Scn8a* gene expression is prominent in neuropeptide Y (Npy)‐positive cells at the cardiovascular pole of the SG, where the cardiac‐innervating cells are located.^36^ Downregulation of the *Scn8a* gene has previously been shown to reduce sodium current in the rat sensory ganglionic cells and slow neuronal firing.^43^ It could thus be suggested that the observed decreased mRNA expression of *Scn8a* causes neuronal AP alterations through reduced sodium current densities. However, specific channel isoform contributions to the voltage‐gated sodium currents in the loose patch clamp were not separated. The sodium blocker used in this study was a pan‐sodium blocker; therefore, the specific contribution of the Nav1.6 channel to sodium current formation remains unresolved. In murine cochlear spiral ganglion, the Nav1.6‐specific blocker 4.9‐ah‐TTX reduced the amplitude of sodium‐activated currents during depolarization by 70%, suggesting a similarly significant contribution of the Nav1.6 channel in forming sodium currents in the ganglionic neuron.^44^ Considering that Nav1.6 channels are abundantly expressed in the SG, it is necessary to assess the contribution of Nav1.6 in forming sodium currents in the SG.^36^ Intriguingly, *Scn8a* has been associated with the maturation of neonatal neuronal development.^45^, ^46^ García et al. reported that there was a postnatal increase in sodium current density in mouse primary cultured motoneurons, attributed to *Scn8a* gene expression.^45^ Furthermore, the Nav1.6 channel expression rapidly increases relative to other sodium channels after birth, whereby it becomes highly expressed in the mouse hippocampus within 30 postnatal days.^46^ Deficiencies in *Scn8a* expression are related to infantile epilepsy and developmental delays in humans.^47^, ^48^ This evidence supports that developmentally regulated *Scn8a* gene expression can impact sodium currents.

The present study examined ionic currents in neurons within in situ ganglia. This was permitted by adopting a loose patch clamp approach applicable to cells within relatively intact tissue. In contrast, whole‐cell patch clamping typically requires isolated cells. Sharp electrodes can access cells in situ, but thus far have been confined to measuring synaptic potentials rather than ionic currents. The latter would require voltage recording and current injection through high‐impedance electrodes in these relatively small cells. However, the loose patch clamping technique also has limitations; it does not provide intracellular access. The loose patch contacts the cell exterior and influences only the membrane area opposite the pipette tip by varying the intrapipette as opposed to the intracellular potential. It, therefore, cannot influence the membrane potential in the cell as a whole, whether in voltage or current clamp mode. As such, it cannot examine the more physiological whole‐cell properties, and accordingly focuses on ionic currents through confined areas of the membrane, nevertheless fulfilling the objectives of the present study. Accordingly, studies of the related stellate ganglion cell firing rates in the present in situ preparation would require the development of additional techniques. Similarly, while offering opportunities to study stellate ganglion cells in situ, the present findings provide only observational insights into corresponding Nav1.6 transcriptional changes. Pharmacological agents specific to and that would access Nav1.6 as opposed to all other 10 Nav isoforms are problematic; and only minute quantities of Nav protein would be isolatable from dissected mouse SG for protein chemistry analysis. This confined our available analysis to studying transcript levels. This analysis limits full details of changes in the underlying protein expression, as transcription levels may not directly parallel translation levels. Nevertheless, the presence of large changes in sodium current and observations of altered transcription exclusively in the Nav1.6 isoform constitutes a significant observation prompting future examination albeit in different experimental preparations amenable to such detailed study.

In conclusion, this study introduces a loose patch clamp as a tool for investigating ion current generated by voltage‐gated channels on SG, and it revealed electrophysiological changes in the SG between the two different aging time points. We believe this study broadens our understanding of the electrophysiological nature of the SG as it ages.

## AUTHOR CONTRIBUTIONS

All authors had full access to all data in the study and take responsibility for the integrity of the data and the accuracy of the data analysis. Conceptualization: B.L., C.L.‐H.H., and K.J. Data curation: B.L. and S.A. Investigation: B.L. and S.A. Formal analysis: B.L. and S.A. Resources: C.L.‐H.H., F.E.N.L., and K.J. Writing original draft: B.L. Writing review and edition: C.L.‐H.H., S.A., C.E.E., and F.E.N.L. Visualization: B.L. and S.A. Project administration: C.E.E. Funding acquisition: K.J. Supervision: K.J.

## CONFLICT OF INTEREST STATEMENT

The authors declare that they have no conflict of interest.

### PEER REVIEW

The peer review history for this article is available at https://publons.com/publon/10.1111/nyas.15298.

## Supporting information



Supporting Information

Supporting Information

## Data Availability

The data that support the findings of this study are available from the corresponding author upon reasonable request.
