# Supplementary material for: Age‐dependent reduction in voltage‐gated inward sodium current and Scn8a gene expression in murine stellate ganglia
Source: Ann N Y Acad Sci. 2025 Feb 25;1545(1):91–104. doi: 10.1111/nyas.15298 (PMC11918529; doi:10.1111/nyas.15298)
Supplement: Supplementary file 1 — Supporting Information [file NYAS-1545-91-s001.docx]

**Supporting data for Reduced tissue level voltage-gated inward sodium current and Nav1.6 channel gene expression by ageing time points in murine stellate ganglia.**

Bonn Lee, Shiraz Ahmad, Charlotte E. Edling, Christopher L.-H. Huang, Fiona E.N. LeBeau, Kamalan Jeevaratnam

**
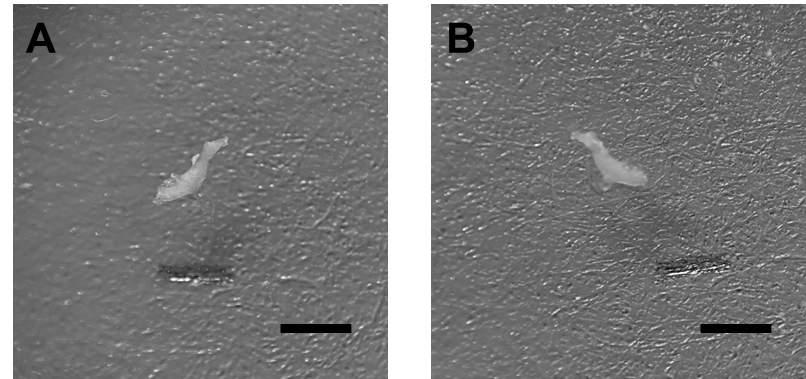
**

**Supplementary figure S1. Tissue preparation for loose patch clamp.** Incubation of the stellate ganglia tissue with collagenase-P. The stellate ganglia tissue was incubated with 20mg/ml collagenase-P dissolved in the artificial cerebrospinal fluid (CSF) solution at 36°C for 10 mins before patched to the pipette, **A**, before incubation, **B**, after incubation, scale bar, 2mm,


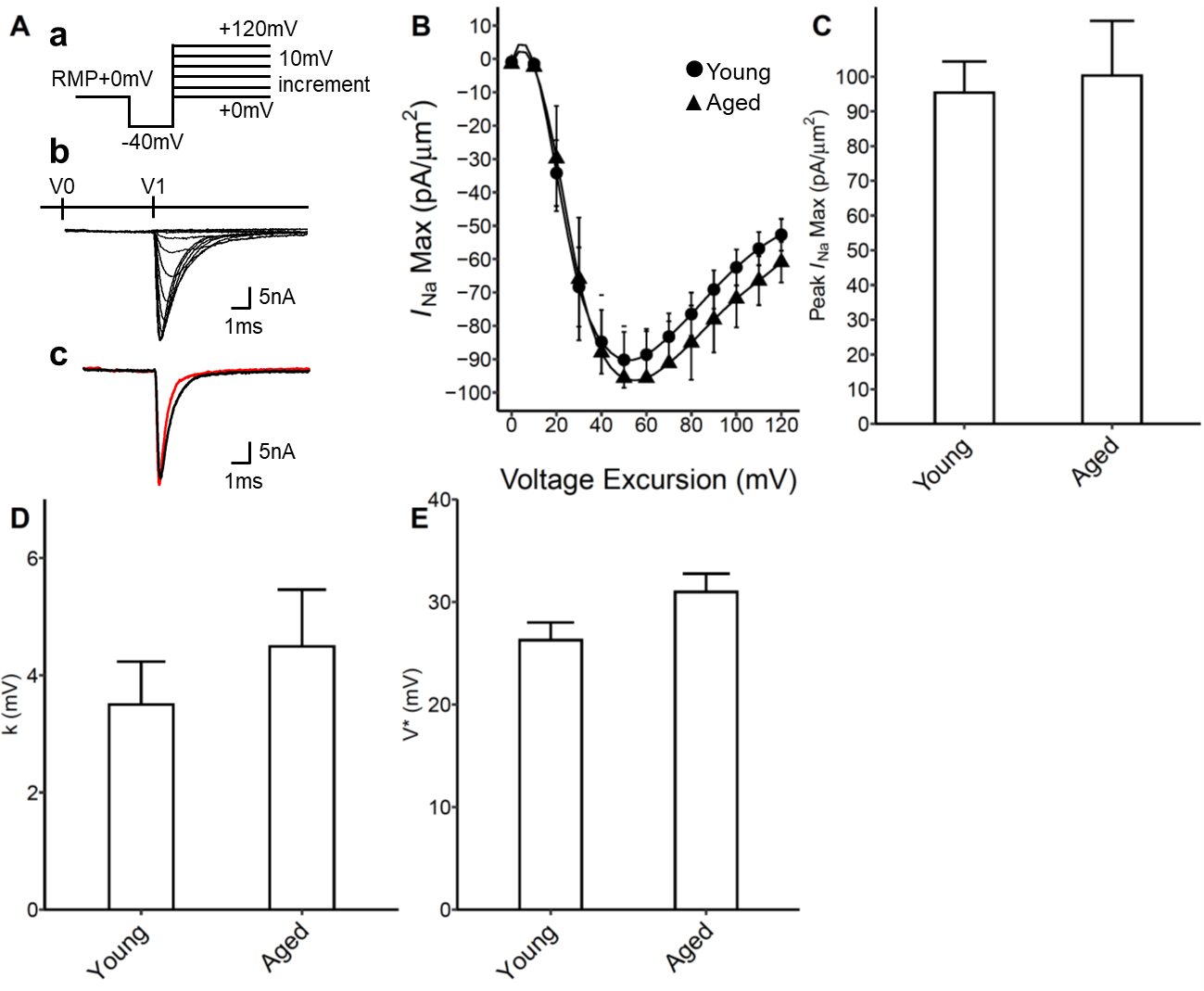


**Supplementary figure S2. Activation properties of voltage-gated inward sodium currents of murine ventricular preparations in two different time points.** **A**, step pulse protocol to activate sodium currents for the ventricular preparations (a), RMP, resting membrane potential, activation pulse protocol began from the RMP, a 4ms duration prepulse was applied to the patched area to remove any residual current over the area of the pipette, then 10ms step-increment pulses were applied to elicit the voltage-gated currents from RMP-40mV to RMP+120mV; family of current traces recorded from the pulse protocol of a (b); the current trace of young and aged ventricular preparation (c); black (young), red (aged); **B**, the current-voltage curve for inward sodium currents, *I*_Na(Max)_ are plotted against the voltage excursion, young ventricle from 4 months-old time point (circle shape, n = 21, from eight independent experiments, 2-3 sites were investigated per tissue), aged ventricle from 13 months-old time point (triangle shape, n = 18, from eight independent experiments, 2-3 sites were investigated per tissue); The *I*_Na_ value increased as the voltage excursion up to its maximum value (*I*_Na(Max)_) on the voltage excursion, and decayed; **C**, the maximum *I*_Na(Max)_ for young and aged ventricle preparations, no statistical significance between the preparation of young and aged time-points of *I*_Na(Max)_ (*p* = 0.067), **D**, The Boltzmann slope factor (*k*) for young and aged ventricle preparation. *I*_Na(Max)_ of 95.40 mV in young, *I*_Na(Max)_ of 100.29 mV in aged. The sodium activation current was fitted with a Boltzmann function, *k* of 4.491mV *V** for I_Na_ in young, 3.498 mV for I_Na_ in aged ventricle preparation, no significance; **E**, the half-maximal voltage (*V**) from a Boltzmann equation described with B, 26.271 mV for *I*_Na_ in young, 30.978 mV for *I*_Na_ in aged ventricle preparation, no significance.

**
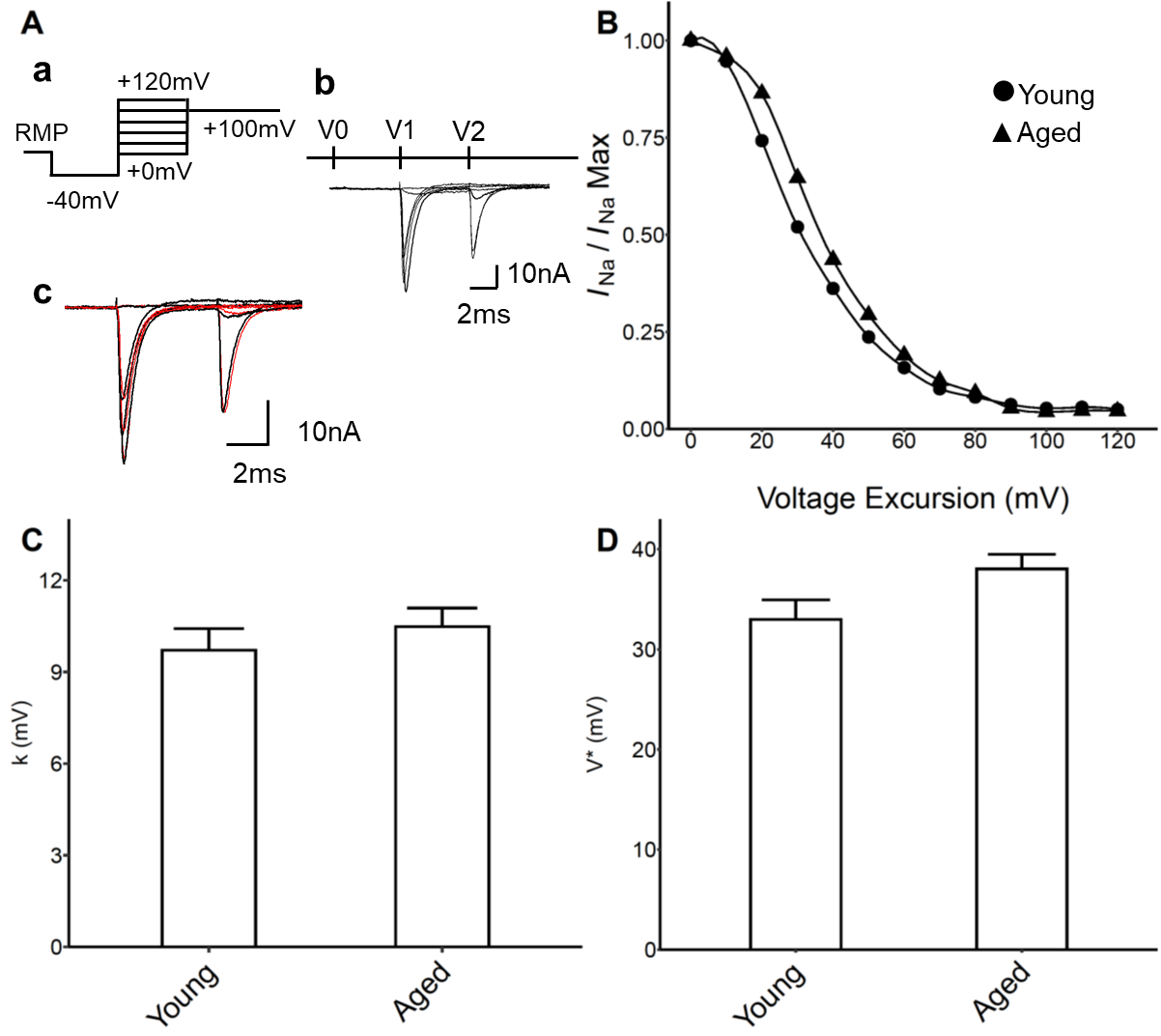
**

**Supplementary figure S3. Inactivation properties of voltage-gated inward sodium currents of murine ventricular preparations in two different time points. A**, step pulse protocol and example current traces. (a) step pulse protocol, RMP, resting membrane potential, activation pulse protocol began from the RMP, a 4ms duration prepulse was applied to the patched area to remove any residual current over the area of the pipette, then 10ms step-increment pulses were applied to elicit the voltage-gated currents negative 40mV from the RMP (RMP-40mV) and to positive 120mV from the RMP (RMP+120mV), finally all sweeps was stopped to a RMP+100mV; (b) current traces from the sodium channel inactivation protocol in a, V0, -40mV prepulse at a 1ms duration; V1, step-increment pulses at a 5ms duration, V2, 100mV pulse at a 10ms duration; (c) example current traces for young and aged ventricular preparation, black trace (young; 4-month-old) and red trace (aged; 13-month-old) ventricular preparation. **B**, the current-voltage curve of sodium channel inactivation under loose patch. *I*_Na_/*I*_Na(Max)_ was plotted against the voltage excursion, each inward sodium current was normalised to the greatest value (*I*_Na(Max)_); Young (circle shape, n = 25, from eight independent experiments, 4-5 sites were investigated per tissue); Aged (triangle shape, n = 25, from eight independent experiments, 4-5 sites were investigated per tissue); **C**, The Boltzmann slope factor (*k*) for ventricle preparation. The sodium inactivation curve was fitted with a Boltzmann function, *k* of 9.71 mV for young ventricle, 10.48 mV for aged ventricle, no significance; D, the half-maximal voltage (*V**) from a Boltzmann equation described with B, 32.98 mV for young ventricular preparation, 38.03 mV for aged ventricular preparation, no significance.


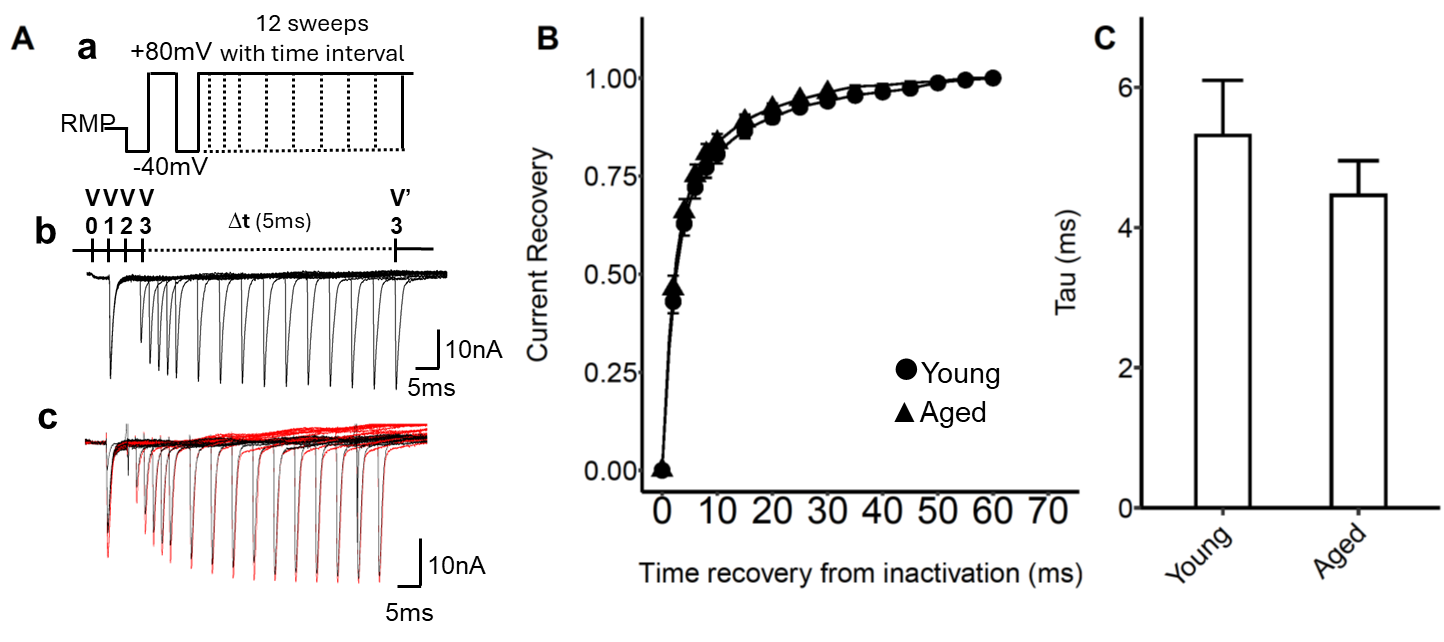


**Supplementary figure S4. Ventricular sodium channel recovery from inactivation following restoration of the membrane potential properties in two different time points.** **A**, the pulse protocol (a), RMP, resting membrane potential, activation pulse protocol began from the RMP, a 4ms duration prepulse was applied to the patched area to remove any residual current over the area of the pipette, then 10ms step-increment pulses were applied to elicit the voltage-gated currents negative 40mV from the RMP (RMP-40mV) and to positive 120mV from the RMP (RMP+120mV), then RMP+100mV was applied; a family of current traces from the sodium channel inactivation protocol in a (b). V0, -40mV prepulse at a 1ms duration; V1, RMP+80mV pulse at a 5ms duration, V2, RMP-40mV pulse at a 10ms duration, V3, RMP+80mV pulse at a different time intervals, Δ*t*, between 5ms (V3) and 65ms (V3’) increment via the 12 successive sweeps making up the protocol. Example current traces for young (4-month-old, black) and aged (13-month-old, red) (c). **B**, the time-voltage curve of sodium channel recovery from inactivation, each *I*_Na_ was normalised to the *I*_Na_ at the termination time-point (65ms), this value was plotted against time intervening between the termination of the conditioning and imposition of the test pulse, young (circle shape, n = 29, from eight independent experiments, 3-4 sites were investigated per tissue), aged (triangle shape, n = 24, from eight independent experiments, 3 sites were investigated per tissue); **C**, the time-constant for young and aged ventricle in the time-recovery from sodium channel inactivation, 5.31ms for young, 4.45ms for young, for aged ventricle, no significance.


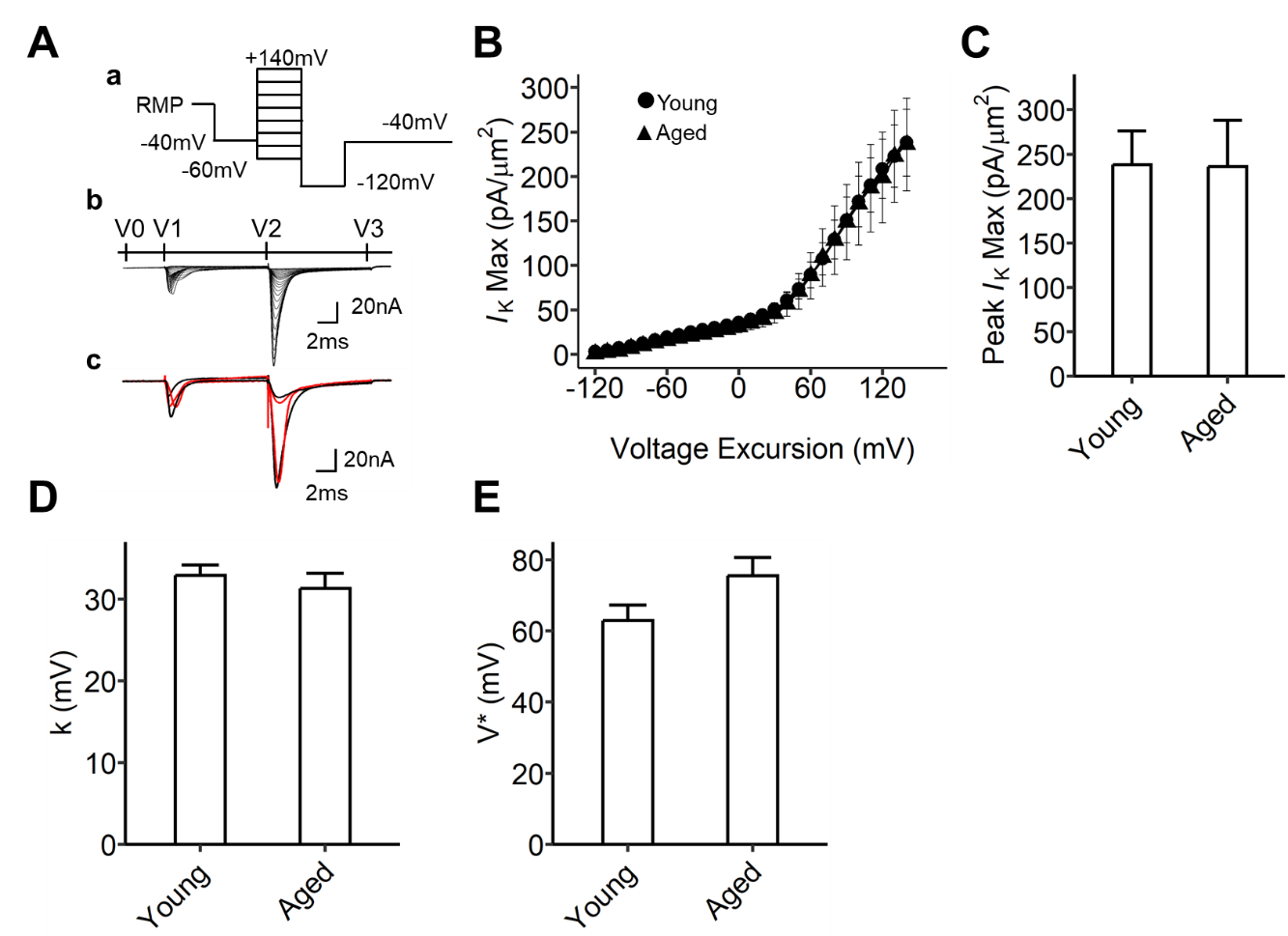


**Supplementary figure S5. Potassium channel activation properties of voltage-gated potassium outward potassium current of murine ventricular preparation in two different time points.** **A**, step pulse protocol to activate potassium channel for the ventricular preparations (a), RMP, resting membrane potential; activation pulse protocol began from the RMP, a 4ms duration prepulse was applied to the patched area to remove any residual current, then a 10ms duration step-increment pulses were applied from RMP+140mV to RMP-60mV through the 21 sweeps, then a 10ms duration hyperpolarising step to RMP-120mV was imposed, finally the membrane potential was turn back to RMP-40mV; a family of current traces from the pulse protocol in a (b). V0, -40mV prepulse at a 1ms duration; V1, -10mV step-increment pulses at a 10ms; V2, hyperpolarising pulse at a 20ms; V3, end pulse at a 30ms. Representative current traces for young (4-month-old, black) and aged (13-month-old, red) (c). **B**, the current-voltage curve of potassium channel activation protocol for ventricle preparation under the loose patch, *I*_k(Max)_ of each current trace was plotted against the voltage excursion; Young (round mark, n = 18, from eight independent experiments, 2-3 sites were investigated per tissue); Aged (triangle, n = 14, from eight independent experiments, 2-3 sites were investigated per tissue); **C**, the maximum *I*_k(Max)_ for the ventricle preparations, 236 mV for young group, 238 mV for the aged group, no statistical significance (*t_27.28_* = -0.032, *p* = 0.974), **D**, The Boltzmann slope factor (*k*) for ventricle preparation. The sodium activation current was fitted with a Boltzmann function, *k* of 32.88mV for *I*_k_ in young group, 31.32mV for *I*_k_ in aged group, no significance (*t*_26.43_ = -0.708, *p* = 0.76); **E**, the half-maximal voltage (*V**) from a Boltzmann equation described with B, 32.93mV for *I*_k_ in young group, 75.54mV for *I*_k_ in aged group, no significance (*t*_31.88_ = -1.88, *p* = 0.068).


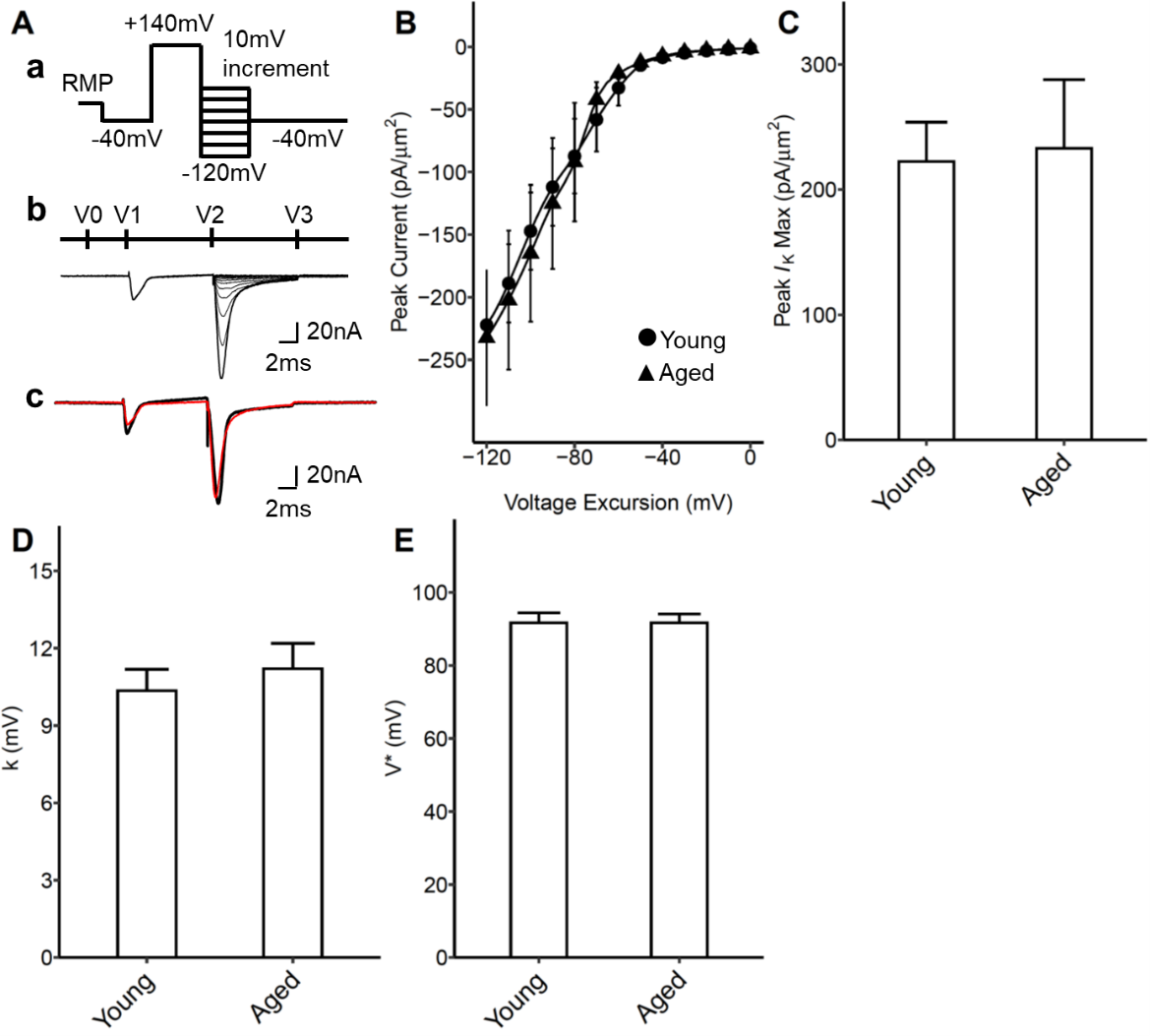


**Supplementary figure S6. Potassium current rectification properties reflected in tail current in ventricular preparations in two different time points. A**, step pulse protocol to rectify potassium current (a), RMP, resting membrane potential; activation pulse protocol began from the RMP, a 4ms duration prepulse was applied to remove residual current over the seal, then a 10ms duration fixed voltage at RMP+140mV were applied, then a 10ms duration voltage sweep from RMP-120mV to RMP+50mV followed, finally the voltage sweep of RMP-40mV was applied; a family of typical current traces from the pulse protocol in a (b). V0, -40mV prepulse at a 1ms duration; V1, 10mV step-increment pulses at a 10ms; V2, hyperpolarising pulse at a 20ms; V3, termination pulse at a 30ms. Representative current trace for young and aged ventricular preparations (c). Young (4-month-old) mice, black trace; aged (13-month-old) mice, red trace. **B**, the current-voltage curve of potassium channel activation protocol for ventricle preparation under the loose patch, *I*_k(Max)_ of each current trace was plotted against the voltage excursion; Young mice (round mark, n = 18, from eight independent experiments, 2-3 sites were investigated per tissue); aged mice, (triangle, n = 14, from eight independent experiments, 2 sites were investigated per tissue); **C**, the maximum *I*_k(Max)_ of ventricle preparations for young and aged mice, -232.93mV for young ventricular preparation, -232.93mV for aged ventricular preparation, no statistical significance (*t26* = 0, *p* = 1), **D**, The Boltzmann slope factor (*k*) for young and aged ventricular preparations. The potassium activation current was fitted with a Boltzmann function, *k* of 11.95mV for *I*_k_ in young heart, 11.07mV for *I*_k_ in aged heart no significance (*t*_27.94_ = 0.29, *p* = 0.51); **E**, the half-max

imal voltage (*V**) from a Boltzmann equation described with B, 41.92mV for *I*_k_ in young heart, 32.43mV for *I*_k_ in the aged heart, no significance (*t*_8.20_ = 1.44, *p* = 0.18).


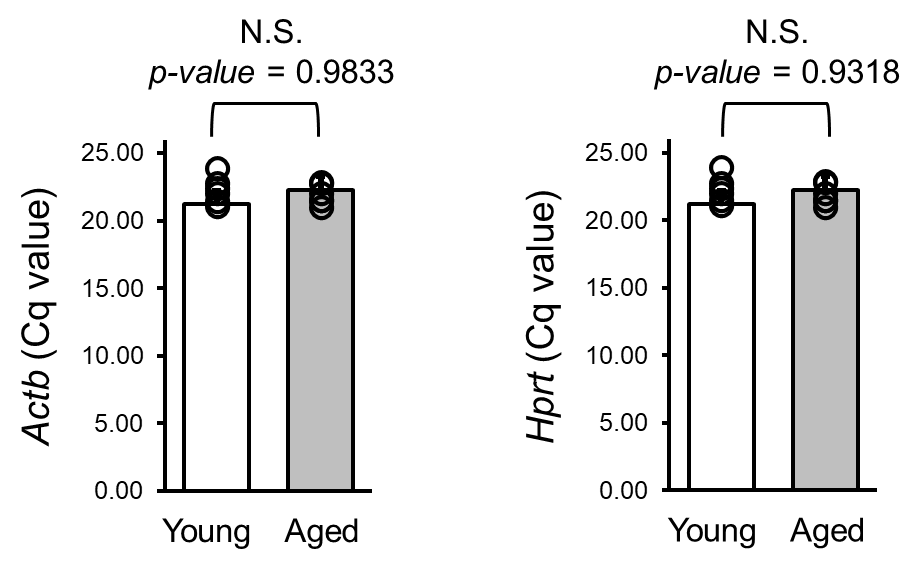


**Supplementary figure S7.** **Stability of the reference housekeeping genes in the stellate ganglia of young and aged mice.** Raw Cq value from the qPCR analysis. *Actb* (left, n = 7 each group), no significance; *Hprt* (right, n = 7 each group), no significance, young mice (4-month-old), aged mice (13-month-old), each data point was overlaid on the bar graph. error bar represents standard deviation (s.d.).

**Supplementary table S1. Oligomer sequences for polymerase chain reaction.**

| Gene | Transcript ID | Description | Forward | Reverse | Product size |
| --- | --- | --- | --- | --- | --- |
| *Scn9a* | NM_001290674 | Sodium voltage-gated channel type 9 alpha subunit 1; Nav1.7 | CCTTGGAACTGGCTGGACTT | CCACGATGGTTTTTAGTCCTGG | 148 |
| *Scn8a* | NM_001077499 | Sodium voltage-gated channel type 8 alpha subunit 1; Nav1.6 | CAACTTCGACAACGTCGGGG | TGCTCGTCCGGCTTTCG | 110 |
| *Scn7a* | NM_009135.2 | Sodium voltage-gated channel type 7 alpha subunit 1; Nav2 | GCCGTGGGAGAATGCAAAAC | TGGGCTGCATGTTTACACCA | 164 |
| *Scn4a* | NM_001403641.1 | Sodium voltage-gated channel type 4 alpha subunit 1; Nav1.4 | GTCATCACAATGGCGTACGTG | TTCACAGACTGGATCAGGGC | 146 |
| *Scn3a* | NM_001355166.1 | Sodium voltage-gated channel type 3 alpha subunit 1; Nav1.3 | TCGCAGATGACAGCCACTTT | CGTAGTTGGGGTTTCGTCCA | 129 |
| *Scn2a* | NM_001099298.3 | Sodium voltage-gated channel type 2 alpha subunit 1; Nav1.2 | TGGGTTGCAGTTCTACATTTCTTG | ACCGTGTTCGAGTTTGACTGG | 149 |
| *Scn1a* | NM_001313997.1 | Sodium voltage-gated channel type 1 alpha subunit 1; Nav1.1 | ACCGTGTTCGAGTTTGACTGG | TCTGGACATTGGCCTGCATC | 119 |
| *ActB* | NM_007393.5 | Beta-actin, housekeeping gene | CGCAGCCACTGTCGAGTC | GTCATCCATGGCGAACTGGT | 96 |
| *Hprt1* | NM_013556.2 | Hypoxanthine phosphoribosyltransferase 1, housekeeping gene | ACAGGCCAGACTTTGTTGGA | ACTTGCGCTCATCTTAGGCT | 150 |
